# Atomic-scale perturbation of oxygen octahedra via surface ion exchange in perovskite nickelates boosts water oxidation

**DOI:** 10.1038/s41467-019-10838-1

**Published:** 2019-06-20

**Authors:** Jumi Bak, Hyung Bin Bae, Sung-Yoon Chung

**Affiliations:** 1 Department of Materials Science and Engineering, Korea Advanced Institute of Science and Technology (KAIST), 291 Daehak-ro, Yuseong-gu, Daejeon, 34141 Korea; 2 KAIST Analysis Center, Korea Advanced Institute of Science and Technology (KAIST), 291 Daehak-ro, Yuseong-gu, Daejeon, 34141 Korea

**Keywords:** Electrocatalysis, Surfaces, interfaces and thin films, Materials for energy and catalysis

## Abstract

A substantial amount of interest has been focused on *AB*O_3_-type perovskite oxides over the past decade as oxygen electrocatalysts. Despite many studies on various compositions, the correlation between the structure of the oxygen octahedra and electrocatalytic property has been overlooked, and there accordingly have been a very limited number of attempts regarding control of atomistic structure. Utilizing epitaxial *Ln*NiO_3_ (*Ln* = La, Pr, Nd) thin films, here we demonstrate that simple electrochemical exchange of Fe in the surface region with several-unit-cell thickness is notably effective to boost the catalytic activity for the oxygen evolution reaction by different orders of magnitude. Furthermore, we directly establish that strong distortion of oxygen octahedra at the angstrom scale is readily induced during the Fe exchange, and that this structural perturbation permits easier charge transfer. The findings suggest that structural alteration can be an efficient approach to achieve exceptional electrocatalysis in crystalline oxides.

## Abstract (web summary)

While perovskite oxides are well-studied for their oxygen electrocatalysis performances, the impact of oxygen octahedra is less studied. Here, authors show electrochemical exchange of lanthanide nickelate surfaces to distort oxygen octahedra, facilitating charge transfer and improving catalysis.

## Introduction

Oxygen evolution and reduction are the major electrochemical reactions in electrolysers, metal-air batteries, fuel cells, and water-splitting devices, and facilitating these reactions has been a central issue for effective fuel generation and higher energy conversion/storage efficiency^1–5^. It is generally accepted that the activation barrier of the oxygen evolution and reduction reactions is fundamentally large and that multiple intermediate steps of electron transfer are necessarily involved during the reactions. Adequate electrocatalysts are thus essential for promoting the reactions at lower overpotentials. In particular, since both the oxygen evolution reaction (OER) and the oxygen reduction reaction (ORR) in metal-air batteries and water-splitting devices take place at room temperature^1,3^, enhancing the oxygen electrocatalysis has a crucial impact not only on the efficiency of such devices, but on their longevity and ultimate performance.

Since the milestone works by Bockris and Otagawa^6,7^ in the early 1980s, substantial attention has been paid to employing perovskite oxides as OER electrocatalysts with the aim of replacing expensive precious-metal-based materials such as IrO_2_ and RuO_2_^3,8,9^. Furthermore, sparked by recent findings on the notably high OER catalytic activity of a multiple-cation perovskite^10^, there has been strong interest in *AB*O_3_-type perovskite oxides as alternative OER catalysts^11–20^. Plausible mechanisms have been reasonably suggested, based on the *B*–OH bond strength (where *B* is a transition metal)^21–23^, the 3*d*-shell electronic configuration^10^, the position of the O 2*p* band center in the electronic band structure^11^, the degree of hybridization between *B*-site metal and oxygen orbitals^14,24^, and the charge-transfer energy between *B* and O^18,25^. Although there is no single universal descriptor for OER electrocatalysis using perovskite oxides^9^, it is recognized in general that the electronic structure of the O 2*p* and metal 3*d* bands strongly correlates with the OER catalytic activity.

A significant change can be induced in the electronic structure of both the metals and the oxygen in many oxides by varying their physical structure, including by lattice strain^26^. Accordingly, one of the crucial initial steps toward establishing the link between the crystal structure, the electronic configurations, and the overall OER catalytic performance is the precise identification of changes in the surface structure by direct observation at the atomic scale^27–29^. From a structural viewpoint, the *AB*O_3_ perovskite framework consists of corner-shared [*B*O_6_] octahedral units, which are catalytically active sites^7^, together with *A*-centered dodecahedra. Consequently, identifying variations in the shape of the [*B*O_6_] octahedra and understanding under what circumstances structural change takes place remain key issues for the accurate prediction of electronic structure and subsequent OER activity. In particular, since the shape of the [*B*O_6_] octahedra is determined largely by the location of oxygen anions in the lattice, visualization of the oxygen positions is an essential aspect of the direct observation of structure, despite being very challenging.

By utilizing (001) epitaxial thin films of lanthanide nickelate perovskites, *Ln*NiO_3_ (where *Ln* is La, Pr, and Nd), in this work we demonstrate that the structural perturbation of metal-oxygen octahedra via simple electrochemical Fe exchange at the surface is particularly efficient at enhancing OER activity, by an order of magnitude. To conduct atomic-column resolved observation of oxygen displacement, we used integrated differential phase-contrast (iDPC) scanning transmission electron microscopy (STEM)^30,31^, a recently developed phase-contrast imaging technique, in addition to well-known annular bright-field (ABF) STEM. Electron energy-loss spectroscopy (EELS) and energy-dispersive X-ray spectroscopy (EDS) were also carried out to verify the chemical exchange of the Fe for the Ni site. Combined with ab initio density functional theory (DFT) calculations, the experimental findings in the present study provide significant evidence showing that a high density of states of O 2*p* and transition-metal 3*d* orbitals near the Fermi level can be achieved by the strong distortion of the oxygen octahedra, boosting the OER activity in the perovskite nickelates.

## Results

### Fe incorporations in LaNiO_3_ thin films

**Fig. 1:**
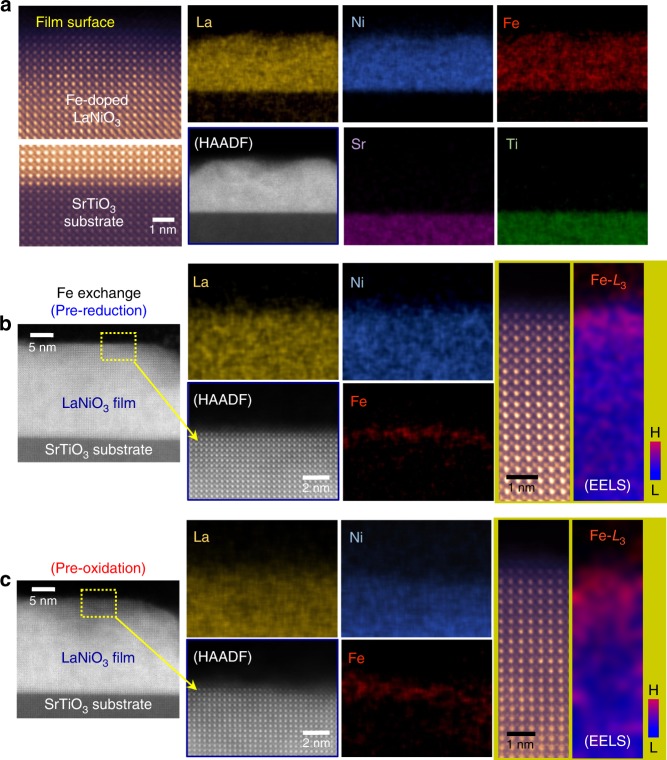
Fe incorporations in LaNiO_3_ epitaxial thin films. All the thin films were fabricated on (001) SrTiO_3_ substrates. **a** The composition of the film is La(Ni_0.9_Fe_0.1_)O_3_. This set of energy-dispersive X-ray spectroscopy (EDS) maps clearly shows the homogenous distribution of Fe over the entire film, verifying a solid-solution. The high-angle annular dark-field (HAADF) image in the left-hand column proves the high crystallinity and epitaxial growth of the film. **b**, **c** These LaNiO_3_ films were subjected to surface Fe exchange by pre-reduction (**b**) and pre-oxidation (**c**) reactions. The presence of Fe in each surface region is confirmed by both EDS and electron energy-loss spectroscopy (EELS) mapping. In particular, the EELS maps and corresponding atomic-column images in the right-hand column show that a detectable amount of Fe is observed down to 3–5 unit cells below the surface

As demonstrated in Ni-based oxides and (oxy)hydroxides, Fe is known to be an effective solute cation that can significantly enhance OER activity^32–41^. We accordingly prepared two different types of (001) thin-film LaNiO_3_ samples for comparison. One was a simple solid-solution homogeneously containing Fe in the lattice by high-temperature annealing during film fabrication. Figure 1a shows a 10%-Fe-doped LaNiO_3_ solid-solution film deposited on a (001) SrTiO_3_ single-crystal substrate. Both the high-angle annular dark-field (HAADF) STEM image and a set of EDS chemical maps in Fig. 1a clearly verify the homogeneous distribution of Fe over the entire film, in addition to epitaxial growth with high crystallinity (see Supplementary Fig. 1 for a series of X-ray diffraction patterns and atomic EDS maps showing the solid-solution of Fe and the epitaxial growth). The other type are LaNiO_3_ thin films that contain Fe exclusively in the surface region, induced via electrochemical ion exchange, which will be described in more detail below. Both sets of EDS maps in the middle column of Fig. 1b, c show the presence of Fe confined to the surface region. Atomic-scale HAADF images also reveal that there is no structural decomposition at the film surface, indicating that the Fe has been substituted for Ni as solute atoms. To more precisely determine the depth of Fe exchange, we obtained electron-energy loss spectrum images using the Fe-*L*_3_ peak during the EELS analysis. Based on the Fe maps shown in the right-hand column of Fig. 1b, c, a distinct amount of Fe is detectable above the background noise from several unit cells below the surface, not merely from the topmost surface (see Supplementary Fig. 2 for two series of Fe-*L*_3_ peaks).

### Surface Fe exchange

**Fig. 2:**
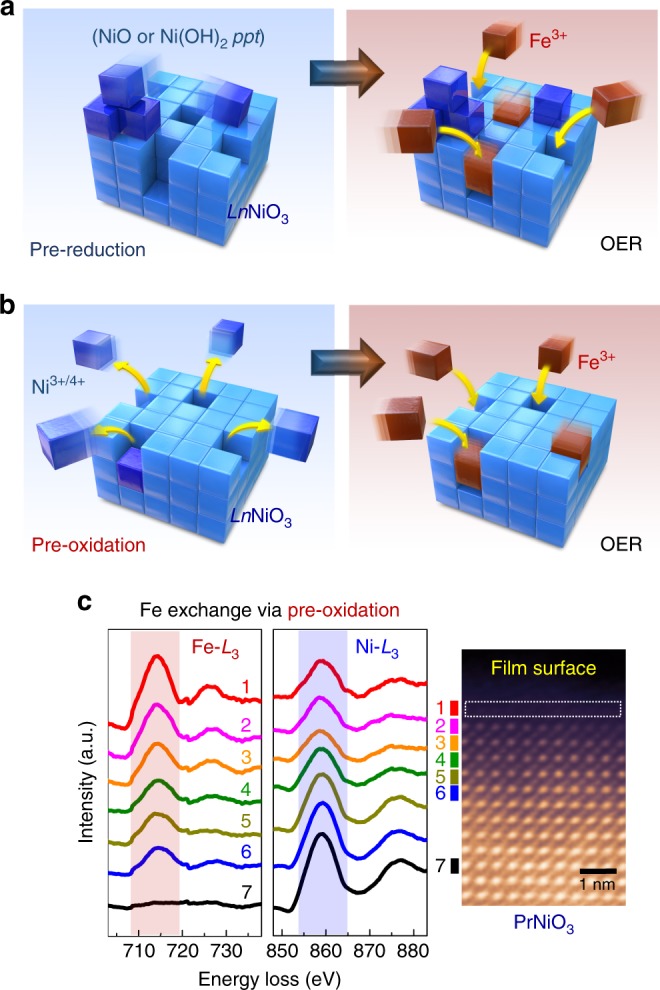
Schematic illustrations and analytical evidence of surface Fe exchange. **a** Pre-reduction reactions are carried out in a 0.1 M KOH aqueous solution in a range of 0.62–1.3 V vs. reversible hydrogen electrode (RHE) so that Ni^3+^ in the nickelate lattice is extracted out. The resulting formation of nanoscale NiO (or Ni(OH)_2_) precipitates is identified. When the film after pre-reduction is oxidized in a 0.1-mM-Fe-containing KOH aqueous solution for measurement of the current density, Fe^3+^ ions in the solution appear to fill the Ni vacancies, resulting in the Fe exchange. **b** A similar procedure occurs during the Fe exchange following the pre-oxidation reaction. Ni dissolution is observed when nickelate thin films are in highly oxidizing conditions (>1.75 V vs. RHE). Subsequently, the Ni vacancies created by the pre-oxidation reactions are filled again with Fe ions from the Fe-containing electrolyte during the oxygen evolution reaction (OER). **c** A layer-by-layer EELS analysis of a PrNiO_3_ film subject to the Fe exchange via pre-oxidation demonstrates the gradual decline in the Fe-*L*_3_ peak intensity and simultaneous increase of the Ni-*L*_3_ peak, directly indicating that Ni is substituted by Fe

The Fe exchanges demonstrated in Fig. 1b, c were achieved by electrochemical methods at room temperature. One approach adopted for the sample shown in Fig. 1b is schematically depicted in Fig. 2a. Ni^3+^ is strongly driven to become Ni^2+^ under reducing conditions in a KOH aqueous solution far below 1.23 V vs. reversible hydrogen electrode (RHE), thereby resulting in the exsolution of Ni from the perovskite lattice. Indeed, when the reduction reaction was repeated many times, we could identify the formation of a seriously Ni-deficient amorphous surface layer, directly demonstrating the Ni extraction (see Supplementary Fig. 3a). At the same time, nanoscale Ni-based oxide (or hydroxide) precipitates, possibly in the form of Ni(OH)_2_ (or NiO) (see Supplementary Fig. 3b) were also found on the surface after 20-cycle reduction reactions down to 0.62 V vs. RHE. As a result, if the number of reactions was adequately controlled at five cycles, Ni vacancies could be created in the surface region without collapsing the crystalline perovskite framework in the surface region. As Ni vacancies are effectively negatively charged defects (*V*_Ni_′′′), electron holes (*h*^•^) confined in the O 2*p* orbitals hybridized with the Ni 3*d* orbitals are anticipated to form in order to satisfy the overall charge neutrality during the pre-reduction reaction (see Supplementary Note 1 for more details, including the Mulliken electronic population analysis). After this pre-reduction, a cyclic potential between 1.27 and 1.75 V vs. RHE was applied to the sample in a Fe-containing KOH solution so that Fe^3+^ would fill the empty Ni sites, as illustrated in Fig. 2a. The high-magnification HAADF image and the Fe EELS map in Fig. 1b demonstrate that this pre-reduction step is very efficient at promoting ion exchange with Fe.

The other approach to achieve Ni extraction is based on oxidation reactions. We experimentally found that Ni cations from LaNiO_3_ thin films could dissolve into a KOH electrolyte solution at high oxidation potentials. Like the previous pre-reduction, a remarkably Ni-deficient amorphous layer could be identified at the film surface after 20-cycle oxidation reactions up to 1.85 V vs. RHE (see Supplementary Fig. 4). As before, when this pre-oxidation step was limited to several cycles to preserve the crystalline framework of the films, Fe exchange could be effectively achieved by filling empty Ni sites with Fe^3+^ from the electrolyte solution. This process is schematically described in Fig. 2b and was experimentally verified by STEM, as shown in Fig. 1c. Oxygen anions in the lattice can evolve at a high overpotential under the oxidizing condition, resulting in the formation of oxygen vacancies. It is thus reasonably anticipated that the negatively charged Ni vacancies (*V*_Ni_′′′) originating from the Ni dissolution are compensated by the positively charged oxygen vacancies (*V*_O_^••^) as Schottky-type defects during the pre-oxidation reaction (see Supplementary Note 2 for more details). Additional evidence obtained by EDS showing the surface Fe exchange through the pre-reduction and the pre-oxidation steps is provided in Supplementary Fig. 5.

As the Ni-*L*_3_ peak in EELS considerably overlaps with the La-*M*_4_ peak, it is fairly difficult to track variations in the Ni-*L*_3_ peak intensity in LaNiO_3_ without rigorous deconvolution of the two peaks^42^. Although a detectable amount of Fe was clearly probed ~3 unit cells beneath the surface in Fig. 1b and ~5 unit cells in Fig. 1c using EELS, it was not possible to verify the change in Ni content in the LaNiO_3_ thin films. We therefore fabricated epitaxial thin films of other nickelates, PrNiO_3_ and NdNiO_3_, where the Ni-*L* peaks do not overlap with Pr- and Nd-*M* peaks, on (001) SrTiO_3_ substrates (see Supplementary Fig. 6 for details on the films). Figure 2c shows two series of Fe-*L* and Ni-*L* peaks acquired in the epitaxial PrNiO_3_ film following Fe exchange via the pre-oxidation method. As denoted by numbers in the atomic-scale HAADF image in the right-hand column, each peak was collected approximately at a unit-cell interval. The two sets of EELS peaks demonstrate the increase in Ni intensity and a simultaneous decrease in Fe intensity with the peak collection below the surface. This directly proves the replacement of Ni with Fe. Consistent sets of Fe-*L* and Ni-*L* peaks obtained in the epitaxial NdNiO_3_ film are also provided in Supplementary Fig. 7.

### Comparison of OER activities and electrochemical impedances

**Fig. 3:**
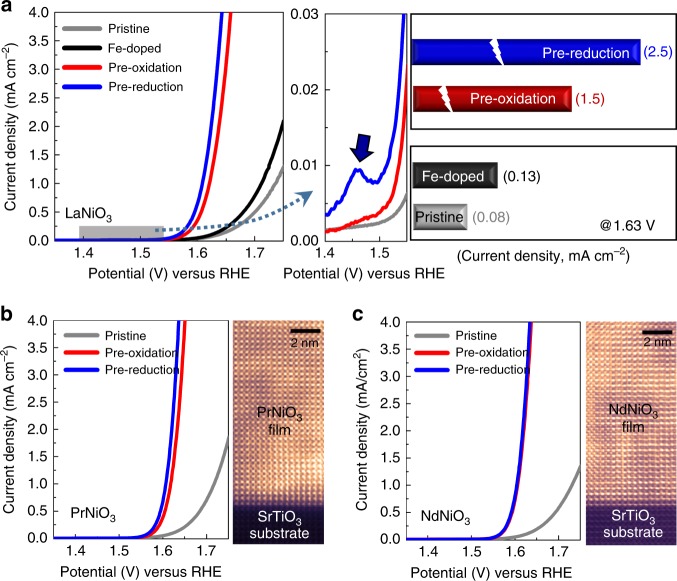
Comparison of OER current densities. **a** Remarkably high OER activities were obtained in the LaNiO_3_ films following the surface Fe exchange that occurred with the pre-reduction and pre-oxidation reactions. Note the much larger OER current values of the surface-Fe-exchange films compared with the current value of the La(Ni,Fe)O_3_ solid-solution film, as specifically compared in the bar graph. The small peak indicated by a black arrow in the blue curve in the middle column represents the occurrence of the Ni^2+^/Ni^3+^ oxidation reaction. **b**, **c** Remarkably enhanced OER activities were consistently identified in both PrNiO_3_ and NdNiO_3_ films following the surface Fe exchange. HAADF images also demonstrate the epitaxial growth of the films along with high crystallinity

The plots in Fig. 3a compare the OER current densities of the different types of thin-film LaNiO_3_ samples, which are pristine (gray), 5%-Fe-doped (black), and Fe-surface-exchange (blue and red). Five percent Fe was selected for the Fe-doped thin-film because this level of doping was observed to show the highest OER activity among the Fe-doped LaNiO_3_ films (see Supplementary Fig. 8 for details). The most important feature readily recognized in Fig. 3a is the exceptionally high OER current densities for the samples with the surface Fe exchange. In particular, as is apparent in the specific comparison in the bar graph in the right-hand column, the OER catalytic activity of the Fe-exchange samples is nearly an order of magnitude higher than that of the Fe-doped solid-solution film. This strongly implies that there is another crucial contribution in addition to the Fe addition. To compare the OER activities of other Fe-containing perovskite oxides with those of our thin-film samples, we also provide an additional bar graph in Supplementary Fig. 9 based on the results of previous reports.

This same significantly higher activity following the ion exchange was also consistently found in both the PrNiO_3_ and NdNiO_3_ epitaxial films, as demonstrated in Fig. 3b, c, respectively. Therefore, the electrochemical Fe exchange appears to be a very effective way of achieving high OER activity in nickelate perovskites in general. As denoted by an arrow in the middle panel in Fig. 3a, the appearance of a small Ni^2+^/Ni^3+^ anodic peak between 1.45 and 1.5 V in the pre-reduction sample confirms the presence of NiO on the surface. This is in good agreement with the schematic illustration shown in Fig. 2a and the EDS results in Supplementary Fig. 3b (see Supplementary Fig. 10 for verification of the anodic peak in other pre-reduction nickelate films). The Tafel plots as well as the values of the Tafel slope for each nickelate are also provided in Supplementary Fig. 11.

**Fig. 4:**
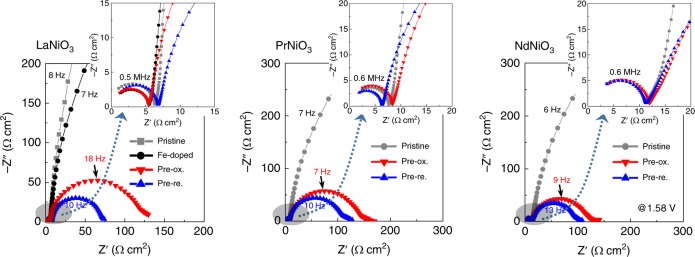
Electrochemical impedance spectroscopy. In contrast to the low-frequency arcs of pristine films, considerably small arcs with very short diameters are noted for the surface-Fe-exchange films, and as a common feature in all three nickelates, indicating remarkably low charge-transfer resistance (*R*_CT_) between the films and electrolytes. As can be seen in each magnified inset for the high-frequency range, denoted by a gray shadow, the electrolyte resistance (*R*_E_) values hardly differ between the samples in each of the nickelates

Electrochemical impedance spectroscopy (EIS) was carried out to examine the electrical charge transfer behavior between the thin films and electrolyte solutions. Figure 4 shows three sets of Nyquist plots obtained by EIS for the LaNiO_3_, PrNiO_3_, and NdNiO_3_ thin films. As these nickelates are metallic^43^, the diameter of the first semicircle in the high-frequency range, which corresponds to the uncompensated resistance (*R*_u_) between the working electrode and the reference electrode^44^, is very short in all three nickelates, showing *R*_u_ merely at an ohm·cm^2^ scale, as demonstrated in each of the insets (see Supplementary Fig. 12 for more details on the high-frequency arcs). Moreover, the *R*_u_ does not significantly vary, irrespective of whether the surface ion exchange has been conducted. In stark contrast, the thin-film samples following the Fe exchange show a huge difference in the diameter of the low-frequency second semicircle representing the resistance of charge transfer (*R*_CT_) at the interface between the film and the electrolyte^23^. As clearly revealed in each Nyquist plot, this remarkable reduction in *R*_CT_ is observed for all the Fe-exchange samples. This notable decrease in charge-transfer barrier at the interface also agrees very well with the exceptionally high OER activity of the thin films following the surface Fe exchange. While there is no *R*_u_ variation, a substantially lower *R*_CT_ with increasing overpotential (*η*) was also observed in each sample (see Supplementary Fig. 13), resulting in a Butler–Volmer type exponential increase in the OER current density as a function of *η*.

### Direct observations with STEM

**Fig. 5:**
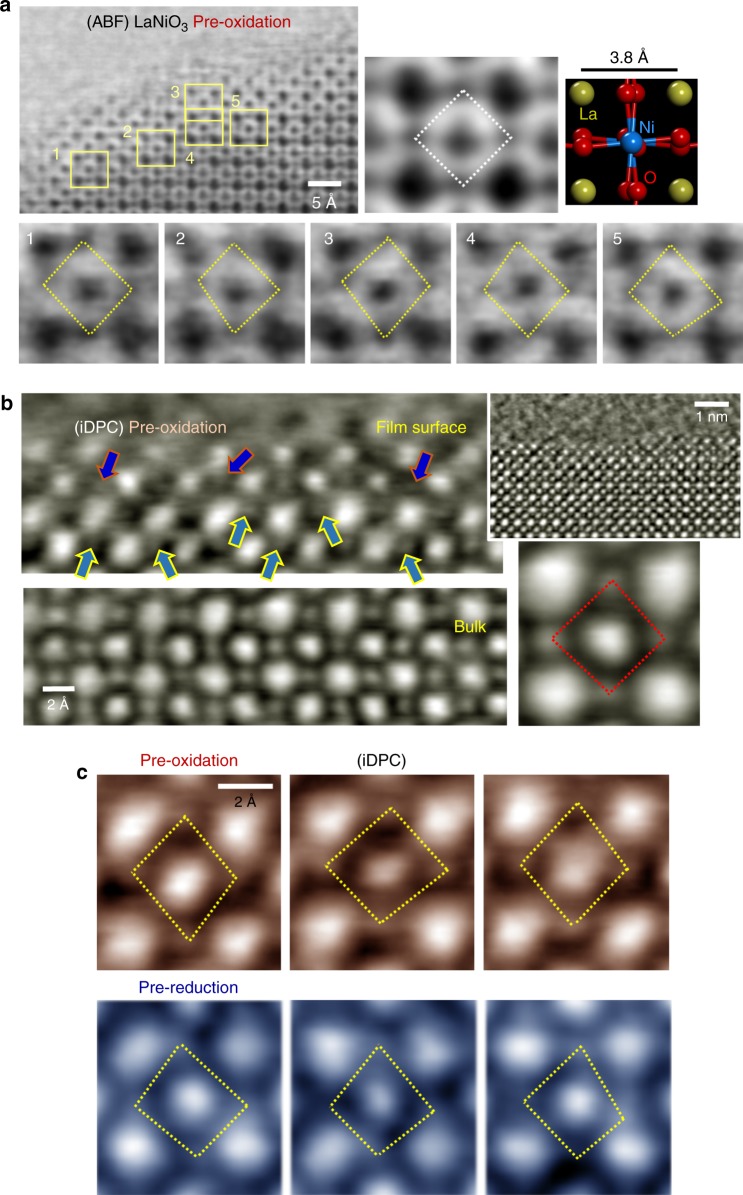
Atomic-scale direct evidence of oxygen-octahedron distortion. The surface region of the LaNiO_3_ films after pre-oxidation and pre-reduction was observed in scanning transmission electron microscopy (STEM). **a** The unit-cell image with the indication of a white diamond was acquired from the bulk region for reference. A series of annular bright-field (ABF) STEM images are provided in the lower panel for the unit cells denoted by a yellow square in the low-magnification image. As indicated by a yellow rhombus, each oxygen octahedron is substantially distorted. **b** Integrated differential phase-contrast (iDPC) STEM shows consistent image features. As indicated by arrows on the image captured from the film surface, serious displacement of oxygen atoms is clarified. **c** iDPC images exemplifying the oxygen displacement are demonstrated for both pre-oxidation and pre-reduction cases

The added Fe clearly has a beneficial influence on the OER catalysis. However, the OER current density of the surface Fe-exchange LaNiO_3_ films was much higher than that of the Fe solid-solution film. This implies that the structural variation that occurs during the Fe exchange reactions may significantly enhance the catalytic activity. Investigating this using STEM at atomic resolution, we observed the surface region of the LaNiO_3_ films after pre-oxidation and pre-reduction. A striking structural feature was identified during the STEM observation: the oxygen anions were seriously displaced from their original positions, resulting in a remarkable distortion of the oxygen octahedra. Figure 5a provides a representative example of the distorted octahedra captured by ABF imaging near the surface of a LaNiO_3_ film after eight cycles of pre-oxidation treatment. A series of magnified ABF images (lower panel) for five unit cells, denoted by yellow squares in the low-magnification image, were compared with the unit-cell image showing four oxygens placed immediately at the corners of a white rhombus for a Ni octahedron in the bulk (upper right panel). The comparison directly shows that most oxygens have been considerably displaced from their original positions in the bulk, as indicated by a distorted yellow rhombus in each of the images (see Supplementary Fig. 14 for the unfiltered raw images). Altogether with the ABF image obtained from the bulk film in Supplementary Fig. 15, an additional set of ABF images for the surface region from the pristine sample without pre-oxidation or pre-reduction is provided in Supplementary Fig. 16 to confirm the undistorted oxygen octahedra as a standard.

ABF-STEM is one of the imaging techniques commonly used for visualizing the position of light elements including oxygen^20,45–50^. However, because atomic columns are imaged in this STEM mode using a dark contrast with a bright background, the position uncertainty of light elements is sometimes not negligible when a specimen is comparatively thick and its tilting angle is high^51,52^. Therefore, to achieve a better signal-to-noise ratio, we used an imaging method, iDPC-STEM, where four-quadrant segmented STEM detectors are utilized for differential phase-contrast image acquisition^30,31^ (see Supplementary Fig. 17 for more details on iDPC-STEM). Figure 5b shows typical iDPC images taken of LaNiO_3_ thin-film samples after the pre-oxidation reactions. In contrast to the well-ordered pseudocubic array of oxygens in the bulk, denoted by a red diamond in the unit-cell image, the arrows in the iDPC image of the surface region directly indicate considerable displacements of oxygen anions. Two series of enlargements along with the indication by yellow rhombuses shown in Fig. 5c provide compelling evidence of the serious distortion of the oxygen octahedra. Based on these two independent STEM imaging methods, it appears that the Ni extraction is inevitably accompanied by perturbation of the oxygen-anion framework during the pre-oxidation and pre-reduction reactions, resulting in serious displacement of oxygens. It is noted that oxygen distortion, in fact, should not be negligible along the *z*-axis, although the images in Fig. 5b merely demonstrate two-dimensional distortions in the *x*-*y* plane. As strong *z*-axis distortion indicates that the displaced oxygen is no longer on the focal plane during the STEM image acquisition, substantially weak column intensity cannot be avoided. As a consequence, it is very likely that *z*-axis distortion of oxygen is responsible for the low intensity of some oxygen columns, for example, in the last image in Fig. 5c.

### DFT calculations

**Fig. 6:**
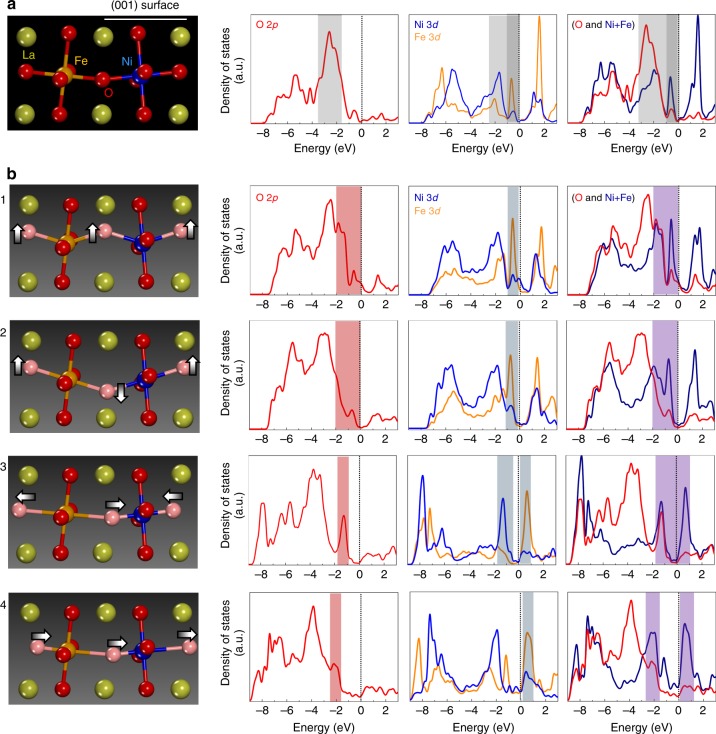
Density of states (DOS) of (001) LaNiO_3_ with Fe. A set of DOS plots for O 2*p*, Ni/Fe 3*d*, and metal/oxygen total states are provided for each case. **a** An optimized supercell of the (001) surface and its DOS are taken as references for comparison. A relatively high density of states in each DOS plot is denoted by a gray shadow. Note that fairly high Fe-3*d* states are identified between −1 and 0 eV below the Fermi level. **b** Four independent cases for oxygen-octahedron distortion are included, showing vertical (cases 1 and 2) and lateral (cases 3 and 4) displacements of oxygen. The significant variations in DOS produced by the oxygen displacements are notable features in the electronic structure. In particular, the substantial increase in the DOS between −2 and +1 eV near the Fermi level has a beneficial effect permitting easier charge transfer between oxygen and metals

Since the first publication of the Jahn-Teller theorem in 1937 (ref. ^53^), it has been widely understood that structural perturbation in transition-metal compounds strongly correlates with changes in the energy state of electrons. Therefore, we carried out ab initio DFT calculations to examine how the density of states (DOS) of the O 2*p* and Ni/Fe 3*d* orbitals in the (001) LaNiO_3_ surface would be affected by the oxygen-octahedron distortion. The atomic illustration shown in Fig. 6a presents part of the (001) surface layer in an Fe-doped LaNiO_3_ supercell after geometric optimization. As denoted by the dark gray shadow in the Ni/Fe DOS in Fig. 6a, one noteworthy aspect regarding the Fe addition to LaNiO_3_ is that the Fe 3*d* states make a substantial contribution to the DOS in the range from −1 to 0 eV, where the number of DOS in the pristine LaNiO_3_ surface is significantly small (see Supplementary Fig. 18a for a set of the DOS plots of the pristine (001) surface without Fe).

More importantly, we identified notable variations in the DOS of both the O 2*p* and Ni/Fe 3*d* orbitals when a subangstrom scale of displacement was introduced in the oxygen anions in each of the supercells. Figure 6b shows four different cases of oxygen displacements and corresponding DOS plots of O 2*p* and Ni/Fe 3*d* levels, demonstrating vertical (cases 1 and 2) and lateral (cases 3 and 4) displacements of oxygens (light pink spheres) with neighboring Ni (blue sphere) and Fe (orange sphere). Specific values for each of the oxygen displacements are provided in Supplementary Fig. 19. Compared with the DOS of the (001) surface shown in Fig. 6a, several significant features can be recognized in these sets of DOS with the oxygen displacements. First, as denoted by the red and blue shadows in the O 2*p* and Ni/Fe 3*d* DOS plots, respectively, there is a considerable increase in electronic states in the range from −2 to +1 eV. For example, a much higher density of the O 2*p* states between −2 and 0 eV is easily observed in cases 1 and 3. A rise in the Fe 3*d* states between −1 and 0 eV caused by the vertical shift of oxygens (cases 1 and 2) is another feature that highlights the effect of oxygen displacements. Further, cases 3 and 4 demonstrate that the lateral shift of oxygens can lead to a substantial increase in the Fe 3*d* states between 0 and +1 eV above the Fermi level, while also contributing to the increase in the Ni 3*d* states between −2 and −1 eV. This high density of Fe 3*d* states near the Fermi level appears to be more dominant compared with the Ni-3*d* DOS variation induced by the oxygen displacements without Fe, as shown in Supplementary Fig. 18b.

## Discussion

Although various factors are involved in oxygen electrocatalysis performance, the charge transfer between transition metals and oxygen appears to be one of the more critical steps. Indeed, as rate-limiting reactions with large activation barriers are usually involved in the formation of intermediate adsorbates of OH* and O* during the OER, facilitating the transfer of electrons between transition metals and adsorbates via oxygen is an important issue^18,25,54,55^. The charge transfer from transition metals to adsorbates via oxygen (e.g., Ni^3+^-O(OH*)^−^ → Ni^4+^-OO*^2−^) would be much easier and more likely to occur if a larger number of density of O 2*p* and metal 3*d* bonding states were placed near the Fermi level rather than far below it. Consequently, as has already been suggested in previous reports, an O 2p band center with a higher position and a higher degree of covalency between metals and oxygen are both beneficial for the OER. Similarly, electron transfer from the O 2*p* band to the empty metal 3*d* band (e.g., Ni^4+^-O^2−^ → Ni^3+^-O(OH*)^−^) also can be remarkably facilitated if a large number of metal 3*d* antibonding states are present as close as possible to the Fermi level, so that the charge transfer energy (the energy difference between the unoccupied Ni 3*d* and occupied O 2*p* band centers) is drastically reduced. In this regard, our DFT calculations for the series of DOS in Fig. 6 directly demonstrate that the distortion of oxygen octahedra is a very effective approach for improving charge transfer between transition metals and oxygen. These results are also in excellent agreement with the exceptional order-of-magnitude improvement in the OER activity of the Fe-exchange samples.

Although more rigorous and extensive investigations are required to precisely understand the changes in DOS induced by the distortion of octahedra, the Jahn-Teller theorem can be applied to qualitatively explain this notable change. Each of the degenerate *t*_2g_ (*d*_xy_, *d*_yz_, and *d*_zx_) and *e*_g_ (*d*_x_^2^-_y_^2^ and *d*_z_^2^) orbitals discreetly splits under the cubic symmetry of oxygen ligands, constructing an equally degenerate upper *e*_g_ level and a triply degenerate lower *t*_2g_ level. However, when the ligands are strongly distorted, such degeneracy is significantly removed in each level, thereby resulting in the nondegenerate five *d* orbitals with various splitting energy gaps. Therefore, the energy range of the bonding states below the Fermi level can be widened, as can be seen in cases 3 and 4 in Fig. 6b. At the same time, a higher DOS may be induced near the Fermi level as a consequence of the narrower gap between the bonding and antibonding states, as denoted by the blue shadows in the Ni/Fe 3*d* DOS plots in Fig. 6b.

Many previous studies on the OER catalysis of oxides have attempted to relate the observed variation in OER activity with the electronic structure of the bulk and the structural change of the overall polycrystals, even though there is substantial crystallographic anisotropy in the catalytic activity^13,56^, which strongly depends on both electronic and atomic structures near the surface. To move past these previous limitations, our study employed a combination of (001) epitaxial thin films, atomic-scale structure and composition analyses, and DFT calculations using more realistic supercells from direct observation. We were thus able to reliably demonstrate a solid correlation among the structural perturbation, the electronic structure, and the resultant OER property in nickelate perovskites. In addition to providing evidence that the Fe exchange has a rather general impact on the exceptional increase in OER activity in nickelate perovskites, the findings in the present work show that symmetry-breaking configurational control of atoms on the surface can offer an important platform toward exceptional oxygen electrocatalysis in perovskite oxides.

We have demonstrated that Fe exchange via selective Ni extraction in the surface region of (001) thin-film nickelate perovskites by electrochemical oxidation and reduction reactions is a remarkably efficient method of achieving exceptional OER activity. In particular, we probed the strong distortion of oxygen octahedra induced by the Ni extraction during the pre-oxidation and pre-reduction steps. A systematic series of DFT calculations showed that this atomic-scale structural perturbation resulted in a significant variation in the O 2*p* and Ni/Fe 3*d* states, especially near the Fermi level, leading to much easier charge transfer between metals and oxygen. Our work highlights the impact of symmetry-broken oxygen geometry on electronic structure and the resulting oxygen electrocatalysis in perovskite oxides.

## Methods

### Thin-film fabrication

Epitaxial *Ln*NiO_3_ (*Ln* = La, Pr, Nd) thin films were fabricated by using a sol-gel process. First, La(NO_3_)_3_·6H_2_O (99.999%, Aldrich), Pr(NO_3_)_3_·6H_2_O (99.99%, Aldrich), Nd(NO_3_)_3_·6H_2_O (99.9%, Aldrich), and Ni(CH_3_COO)_2_·4H_2_O (99.998%, Aldrich) were used as starting materials for the preparation of precursor solutions. Each of the lanthanide nitrates and the nickel acetate were first dissolved in 2-methoxyethanol under a constant stirring condition to prepare precursor solutions with 0.2 M. For complete dissolution of the source materials, refluxing was carried out at 80 °C for 1 h. Each of the precursor solutions was deposited on (001) SrTiO_3_ single-crystal substrates by a spin-coating method at 5000 rpm for 10 s. The wet films were dried at 150 °C for 10 min on a hot plate, subsequently heat-treated at 400 °C for 10 min for pyrolysis, and finally annealed at 800 °C for 1 h in air for LaNiO_3_ films, and at 700 °C for 30 min in an O_2_-flow (400 sccm) atmosphere for PrNiO_3_ and NdNiO_3_ films for crystallization. The epitaxy of the grown films was confirmed by X-ray diffractometry (X’Pert-PRO MRD, PANalytical) with Cu-*K*_α_ radiation and STEM observation.

### Scanning transmission electron microscopy, energy-dispersive X-ray spectroscopy, and electron energy-loss spectroscopy

Samples for STEM observation were prepared by lift out via ion-beam milling in a focused ion-beam system (Quanta 3D FEG and Helios G4 UX, Thermo Fisher Scientific). Protective amorphous carbon and thin Pt layers were applied over the region of interest before milling. To minimize the sidewall damage and sufficiently thin the specimen for electron transparency, final milling was carried out at a voltage of ~2 kV. Conventional STEM images were taken with a transmission electron microscope (Titan cubed G2 60–300, Thermo Fisher Scientific) at 300 kV with a spherical aberration (Cs) corrector (CEOS GmbH). The optimum size of the electron probe was ~1 Å with a convergence semiangle of 19 mrad. The collection semiangles of the STEM detectors were set to 67.6–200 mrad for HAADF imaging and 12.1–67.6 mrad for ABF imaging. iDPC images were obtained using a four-quadrant segmented detector attached in Titan Themis Z (Thermo Fisher Scientific) with a Cs corrector at 300 kV. The obtained raw images were band-pass filtered to reduce background noise. Chemical mapping with EDS was carried out in the Titan cubed G2 at 300 kV along with four integrated silicon-drift EDS detectors (ChemiSTEM™ technology) at a collection solid angle of 0.7 srad. La-*L*_α_ (4.6 keV), Pr-*L*_α_ (5.0 keV), Nd-*L*_α_ (5.2 keV), Ni-*K*_α_ (7.5 keV), and Fe-*K*_α_ (6.4 keV) lines were selected during elemental mapping. The probe current was adjusted to be 50–100 pA with a scanning time of <300 s. The EDS maps were low-pass filtered using Bruker ESPRIT software after the reduction of background noise for better visualization. EELS analysis was performed with a Gatan Image Filter (GIF Quantum 965, Gatan Inc.). Electron energy-loss spectra for the Ni-*L* and Fe-*L* edges were acquired for spectrum imaging with a dispersion of 0.25 eV per channel and a collection aperture of 5 mm in diameter.

### Electrochemical characterization

All electrochemical reactions and measurements were conducted with a potentiostat (Biologic SP-300) in a 0.1 M KOH aqueous solution (pH = 12.9) prepared by using the Milli-Q water (18.2 MΩ cm) and KOH pellets (Sigma Aldrich, 99.99%) to achieve sufficiently high purity. According to the chemical analysis by inductively coupled plasma—optical emission spectrometry (ICP-OES), the Fe impurity in our electrolyte solutions was identified to be less than 5 ppb. Furthermore, when we carried out the oxidation reactions up to 1.85 V several times, we found that the OER current density continuously decreased (see Supplementary Fig. 20), directly demonstrating the absence of an Fe-impurity effect in the electrolyte. A Pt counter electrode and a saturated Ag/AgCl reference electrode were used. The measured potential values vs. the Ag/AgCl reference electrode were converted into the RHE scale by using the following equation at 25 °C,

1 $$E_{{\mathrm{RHE}}} = E_{{\mathrm{Ag}}/{\mathrm{AgCl}}} + 0.059 \cdot {\mathrm{pH}} + E^\circ _{{\mathrm{Ag/AgCl}}}$$

where *E*_RHE_ is the converted potential vs. RHE, *E*_Ag/AgCl_ is the measured potential against the Ag/AgCl reference electrode, and *E*°_Ag/AgCl_ is the standard potential of Ag/AgCl (KCl 3 M) at 25 °C, i.e., 0.21 V. Pre-reduction reactions (up to five cycles) were carried out in a 0.1 M KOH aqueous solution in a potential range from 1.30 down to 0.62 V vs. RHE, which belongs to the stable range of Ni^2+^ (approximately 0.1–1.3 V vs. RHE at pH = 12.9)^57^, at a sweeping rate of 10 mV/s. Pre-oxidation reactions (up to six cycles) were also conducted in an identical manner in a potential range from 1.27 to 1.85 V vs. RHE. For Fe exchange after the pre-reduction or pre-oxidation reactions and simultaneously cyclic potential was applied (ten cycles) to the samples in a range from 1.27 to 1.75 V vs. RHE in a 0.1 M KOH aqueous solution with 0.1 (or 1.0) mM Fe(NO_3_)_3_·9H_2_O so that Ni vacancies created in the surface region of the films could be filled with Fe from the solution (see Supplementary Fig. 21 for the potential range comparison between the pre-reduction, pre-oxidation, and Fe exchange reactions). OER current densities were also simultaneously reordered in the same Fe-containing KOH solution. As reference data for comparison, the OER current densities of pristine and Fe-doped thin-film samples were also measured in the same potential range (1.27–1.75 V vs. RHE) in a Fe-free KOH solution. All electrolyte solutions were presaturated by bubbling O_2_ for 30 min under constant O_2_ bubbling. The substrate and the connecting copper wire were completely covered with chemically inert insulating epoxy resin after application of silver paint on the back side of a thin-film sample so as to expose the film surface only. Electrochemical impedance spectroscopy to investigate both the uncompensated series resistance (*R*_u_) for *iR*_u_ correction of the applied potential and the interface charge-transfer resistance was also carried out in the same potentiostat in a frequency range from 0.1 Hz to 1 MHz with an amplitude of 10 mV. Double layer capacitance was measured to examine the change in comparative surface roughness after the Fe exchange. As previously reported^58^, the capacitance measurements were carried out at 0.05–0.20 V vs. Ag/AgCl. No significant variation in capacitance was identified in each nickelate case, verifying that there was no change in the surface morphology responsible for the exceptional increase of OER activity.

### DFT calculations

Ab initio DFT calculations for DOS variation at the (001) surface of pristine and Fe-doped LaNiO_3_ were carried out using the spin-polarized local density approximation (LDA) functional for exchange correlation, along with the ultrasoft pseudopotentials for ionic cores, as implemented in the CASTEP code (Biovia Inc.). A sufficiently long (001)-surface slab along with a 10-Å vacuum layer was constructed as an optimum supercell for each calculation to make the relaxation layer of each slab more than 10 Å in thickness. To account for the electron localization around Ni and Fe ions, the LDA + *U* method with the Hubbard *U* parameter (4.0 eV for both Ni 3*d* and Fe 3*d* states) was employed^59,60^. Low-spin (*t*_2*g*_^6^)(*e*_*g*_^1^) for *d*^7^ Ni^3+^ and high-spin (*t*_2*g*_^3^)(*e*_*g*_^2^) for *d*^5^ Fe^3+^ configurations were assumed, respectively^61^. The plane-wave basis set for the kinetic energy cutoff was 500 eV. Relaxation of the internal coordinates for each atom was performed using the Broyden–Fletcher–Goldfarb–Shanno (BFGS) algorithm with convergence tolerances of 0.1 eV/Å for the maximum ionic force, 5 × 10^−5^ eV/atom for the total energy, and 0.005 Å for the maximum ionic displacement. The charge variation of each oxygen atom before and after the formation of Ni vacancy could be obtained from the Mulliken electronic population analysis^62,63^.

## Supplementary information

Supplementary Information

## Data Availability

The data that support the findings of this study are available from the corresponding author (S.-Y.C.) upon reasonable request.
