# Supplementary material for: Atomic-scale perturbation of oxygen octahedra via surface ion exchange in perovskite nickelates boosts water oxidation
Source: Nat Commun. 2019 Jun 20;10:2713. doi: 10.1038/s41467-019-10838-1 (PMC6586858; doi:10.1038/s41467-019-10838-1)
Supplement: Supplementary file 1 — Supplementary Information [file 41467_2019_10838_MOESM1_ESM.pdf]

Supplementary Information

Atomic-scale perturbation of oxygen octahedra via surface ion exchange in perovskite nickelates boosts water oxidation

Bak et al.

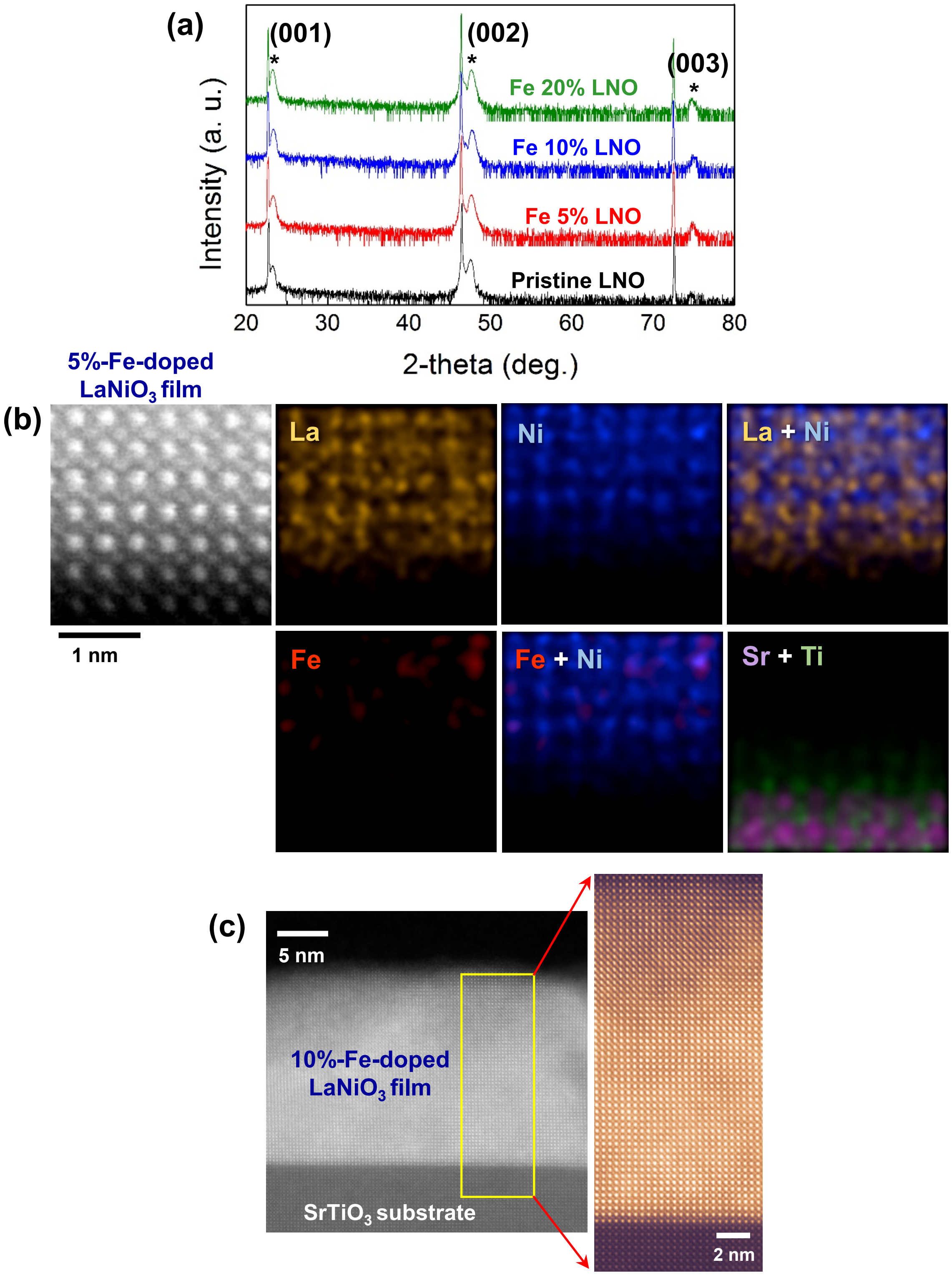

Supplementary Fig. 1. Fe-doped $LaNiO_3$ epitaxial thin films deposited on (001) $SrTiO_3$ substrates. (**a**) As denoted by asterisks, only the (00*l*) Bragg reflections from the film are shown along with the high-intensity substrate $SrTiO_3$ substrate peaks in each of the X-ray diffraction pattern, demonstrating the epitaxial growth of Fe-doped $LaNiO_3$ films with sufficiently high crystallinity. (**b**) Atomic-scale EDS maps together with a HAADF image also support the heteroepitaxial growth of 5% Fe-doped $LaNiO_3$ films on a $SrTiO_3$ substrate in addition to the Fe substitution for Ni. (**c**) In agreement with the X-ray diffraction result, the magnified HAADF image for the region indicated by a yellow rectangle in the low-magnification image directly verifies the (001) single-crystal film of 10%-Fe-doped $LaNiO_3$.

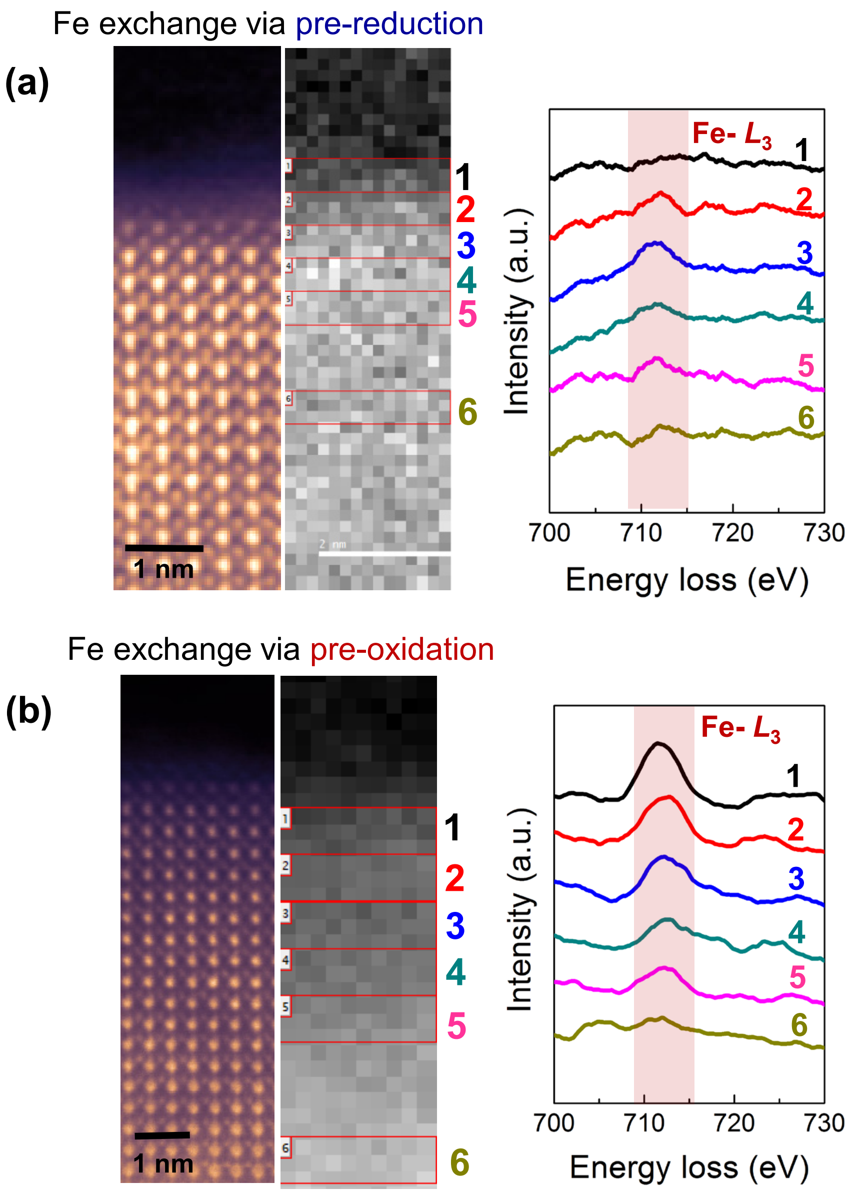

Supplementary Fig. 2. EELS analysis and Fe-L_3 spectra for the surface Fe-exchange thin-film samples. The detectable intensity of the Fe-L_3 peak from spectrum 1 to spectrum 5 in both (**a**) pre-reduction and (**b**) pre-oxidation samples indicates that the Fe exchange takes place several unit cells beneath the surface, in good agreement with the mapping results shown in Figures 1b and 1c in the main text.

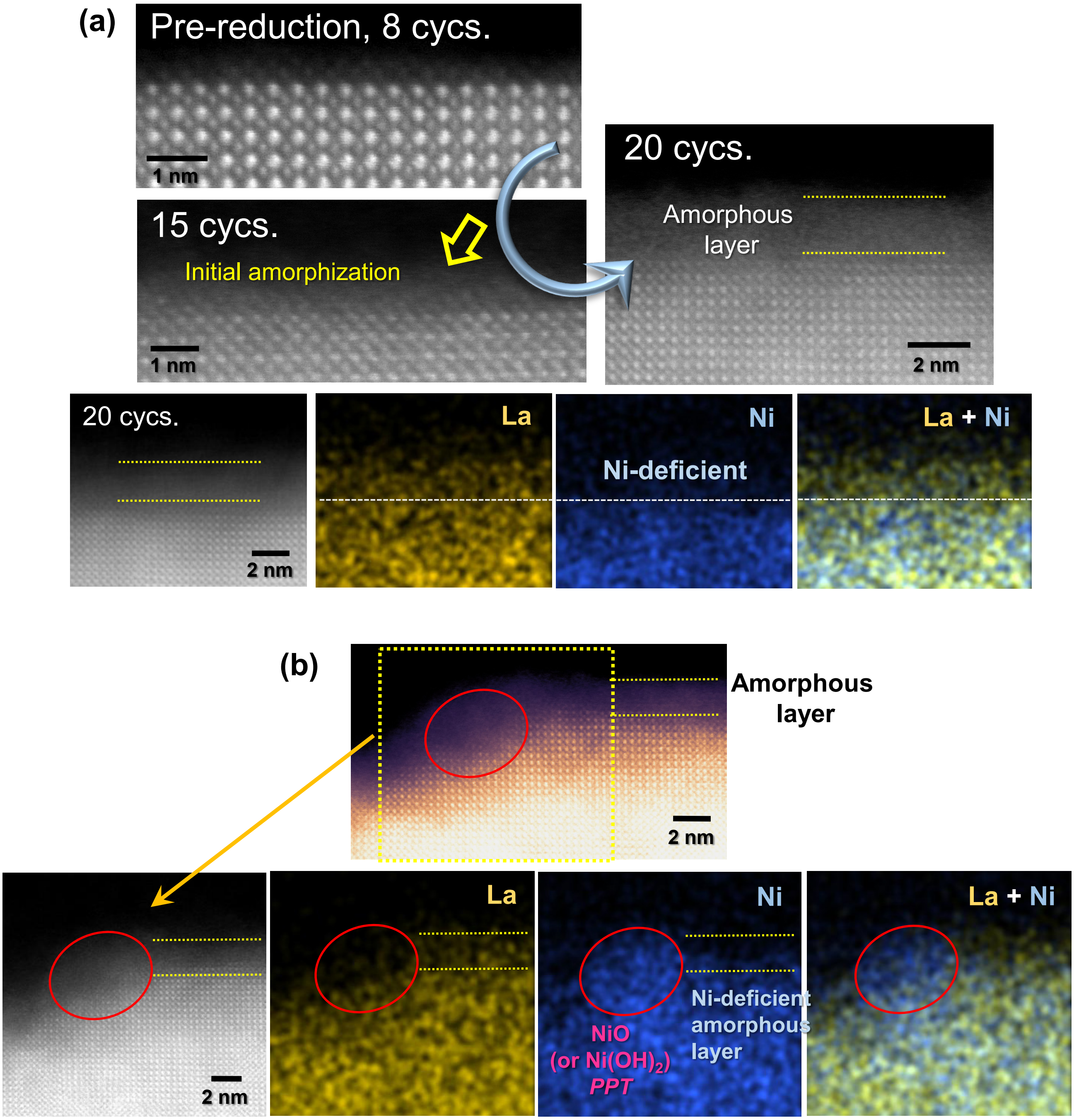

Supplementary Fig. 3. Surface structure evolution by pre-reduction reactions in $LaNiO_3$ films. (**a**) No significant structural variation occurs at the surface during the 8-cycle pre-reduction reactions. However, after 15 cycles amorphization at a few unit-cell scale is initiated, as denoted by a yellow arrow. A nanometer-thick amorphous layer (yellow lines) finally forms over the surface when the pre-reduction is repeated more than 20 times. A set of EDS maps clarifies that the amorphous layer is seriously Ni-deficient, indicating the Ni exsolution produced by the pre-reduction reactions. (**b**) In addition to the formation of a Ni-deficient amorphous layer, Ni-rich oxide precipitates are found on the surface as well. As an example indicated by a red circle, nanoscale NiO secondary precipitates are identified during the EDS analysis, consistently supporting the Ni exsolution.

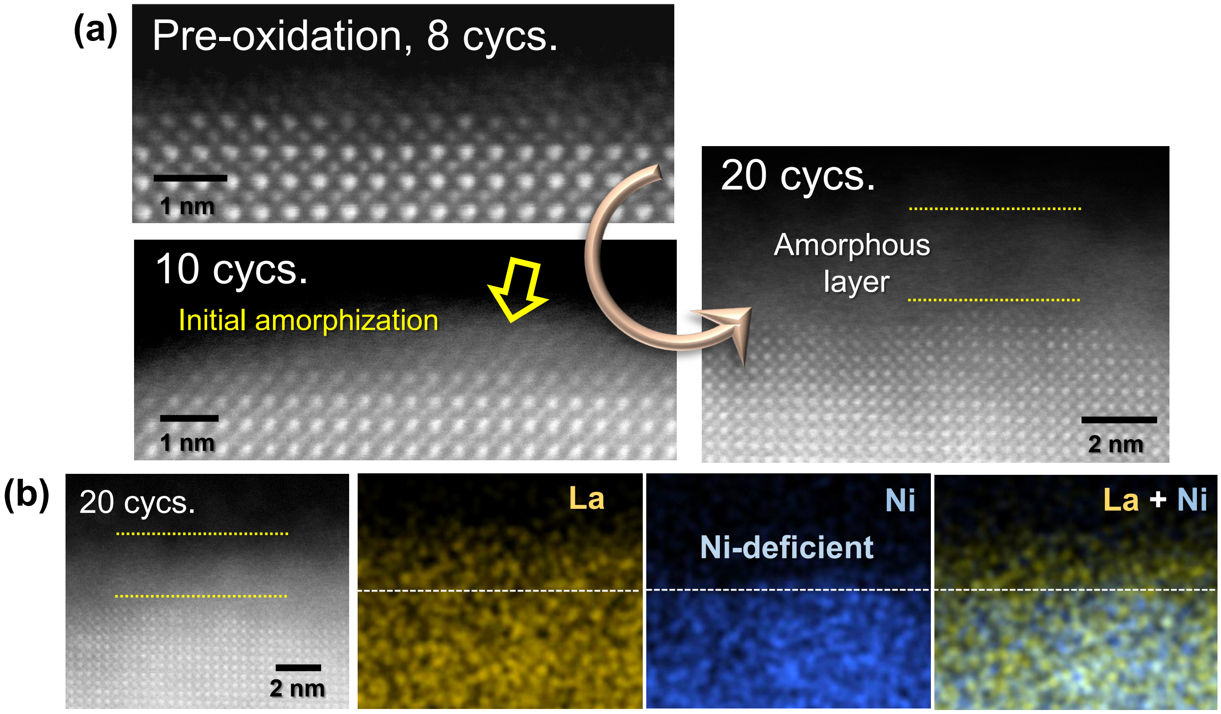

Supplementary Fig. 4. Evolution of surface structure by pre-oxidation reactions in $LaNiO_3$ films. (**a**) In this pre-oxidation case, Ni appears to be extracted at high oxidizing overpotentials and dissolve into an electrolyte solution during the reactions. As indicated by a yellow arrow, a surface amorphous layer begins to form after 10-cycle pre-oxidation reactions. (**b**) The EDS maps for a 20-cycle pre-oxidation sample reveal that the composition of the nanometer-thick amorphous layer is remarkably Ni-deficient, consistent with Ni dissolution from the lattice.

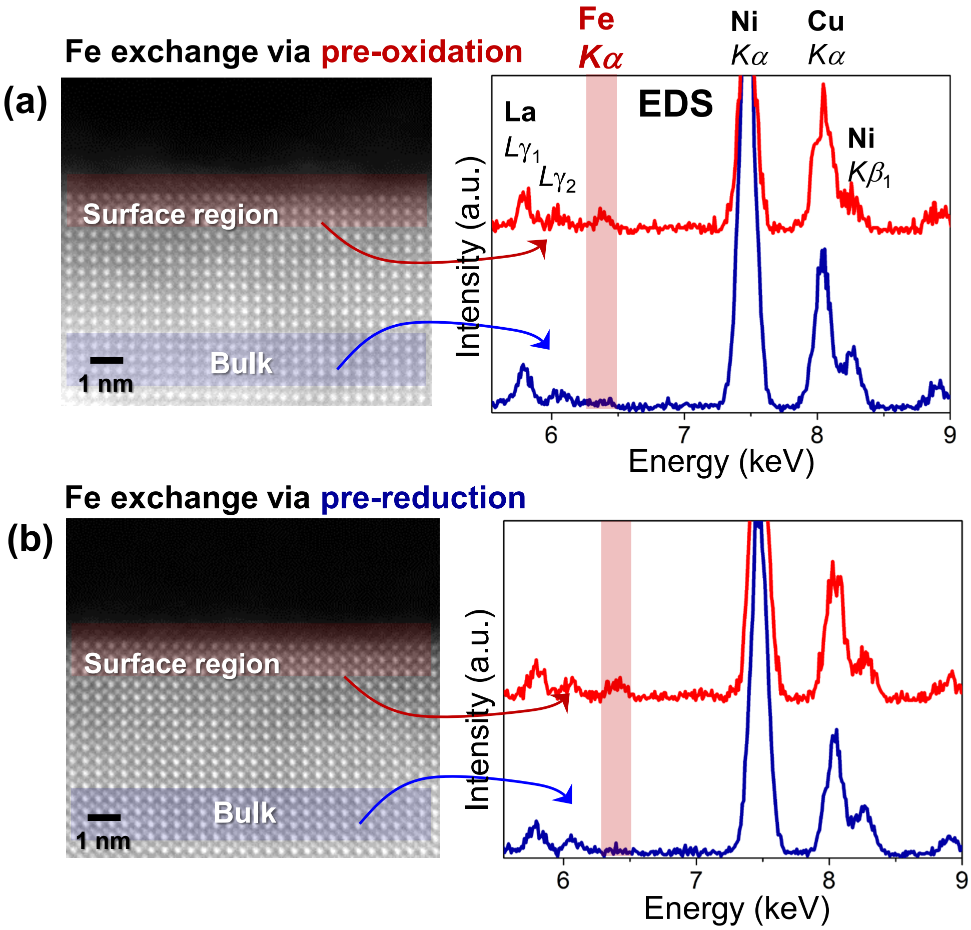

Supplementary Fig. 5. Comparison of EDS spectra between the surface region and the bulk in surface Fe-exchange samples. In addition to the EELS analysis, as well as EDS mapping in the main text, these two sets of EDS spectra along with the atomic-scale HAADF images confirm the Fe exchange at Ni sites via (**a**) the pre-oxidation and (**b**) pre-reduction reactions, with no change in the perovskite framework. As denoted by a red shadow in each plot, a small Fe-K_{α} peak appears in the surface regions only.

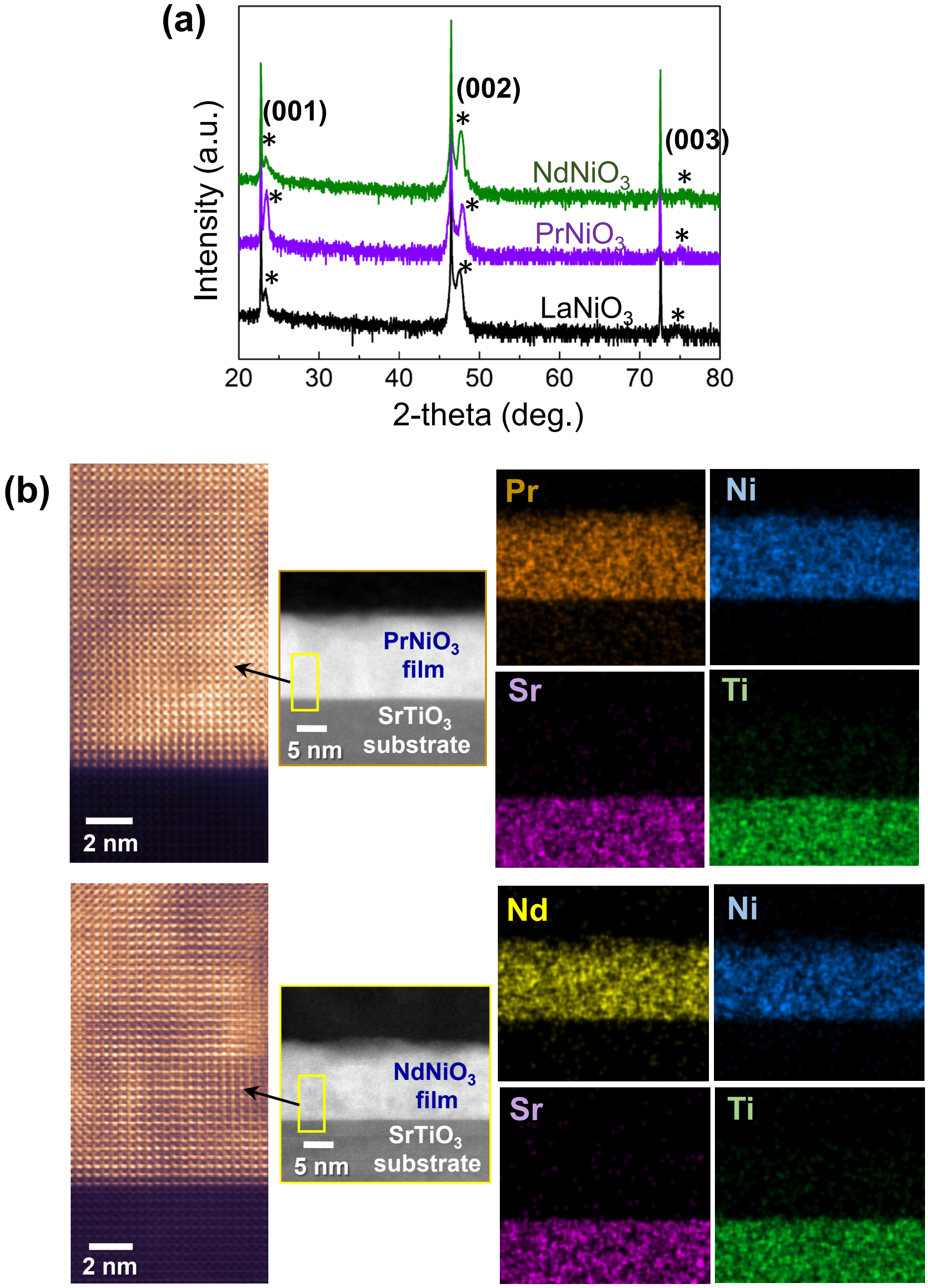

Supplementary Fig. 6. Heteroepitaxial $PrNiO_3$ and $NdNiO_3$ films on $SrTiO_3$ substrates. (**a**) The X-ray diffraction patterns, showing only the (00*l*) Bragg reflections, demonstrate the epitaxy of $PrNiO_3$ and $NdNiO_3$ films deposited on (001) $SrTiO_3$ single-crystal substrates. The diffraction pattern of the $LaNiO_3$ film is also provided for comparison. The high-intensity diffraction peaks originate from the substrate. (**b**) Atomic-level HAADF-STEM images and EDS maps also consistently demonstrate the epitaxial growth of the films with high crystallinity and chemical homogeneity.

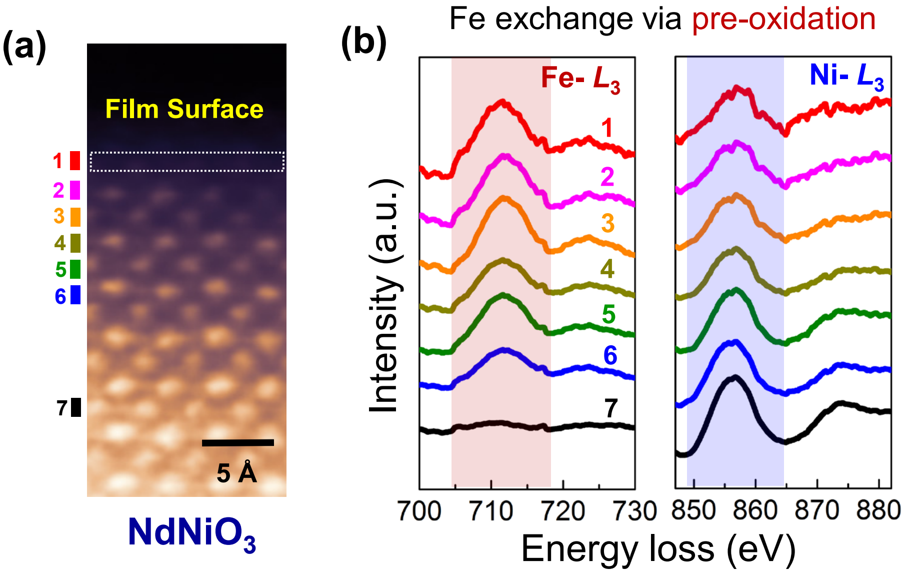

Supplementary Fig. 7. EELS analysis of the Fe exchange in a NdNiO$_3$ film. (**a**) A layer-by-layer (from 1 to 7) EELS analysis was carried out for a NdNiO$_3$ film subjected to the Fe exchange via pre-oxidation. (**b**) The gradual decrease in the Fe-L_3 peak intensity (left) and simultaneous increase in the Ni-L_3 peak (right) are demonstrated, verifying the Fe substitution for Ni.

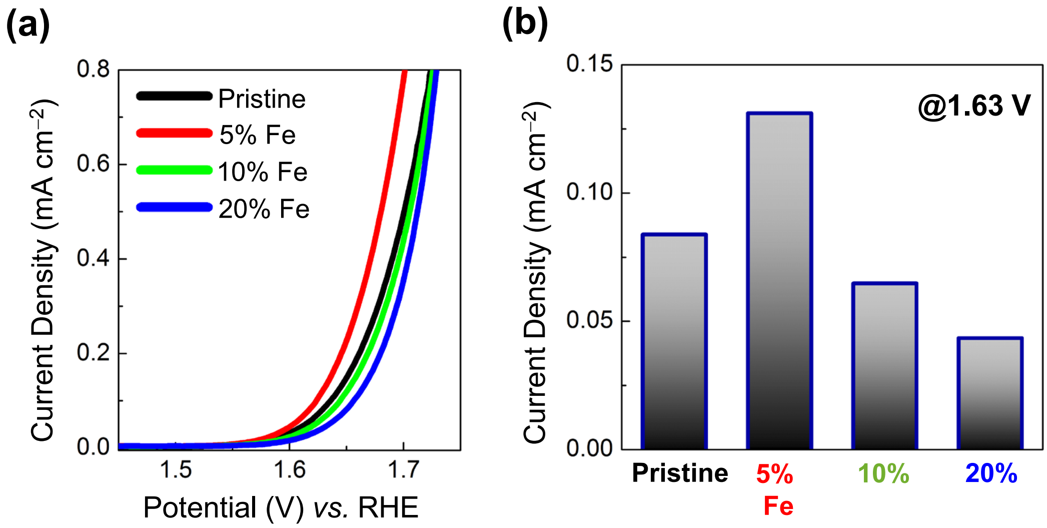

Supplementary Fig. 8. OER activities of Fe-doped (001) $LaNiO_3$ films. (**a**) The highest activity appears when 5% Fe is doped. (**b**) This bar graph compares the OER current densities obtained at 1.63 V *vs*. RHE. Based on this result, 5%-Fe-doped solid-solution films were utilized for all the experimental measurements in the main text.

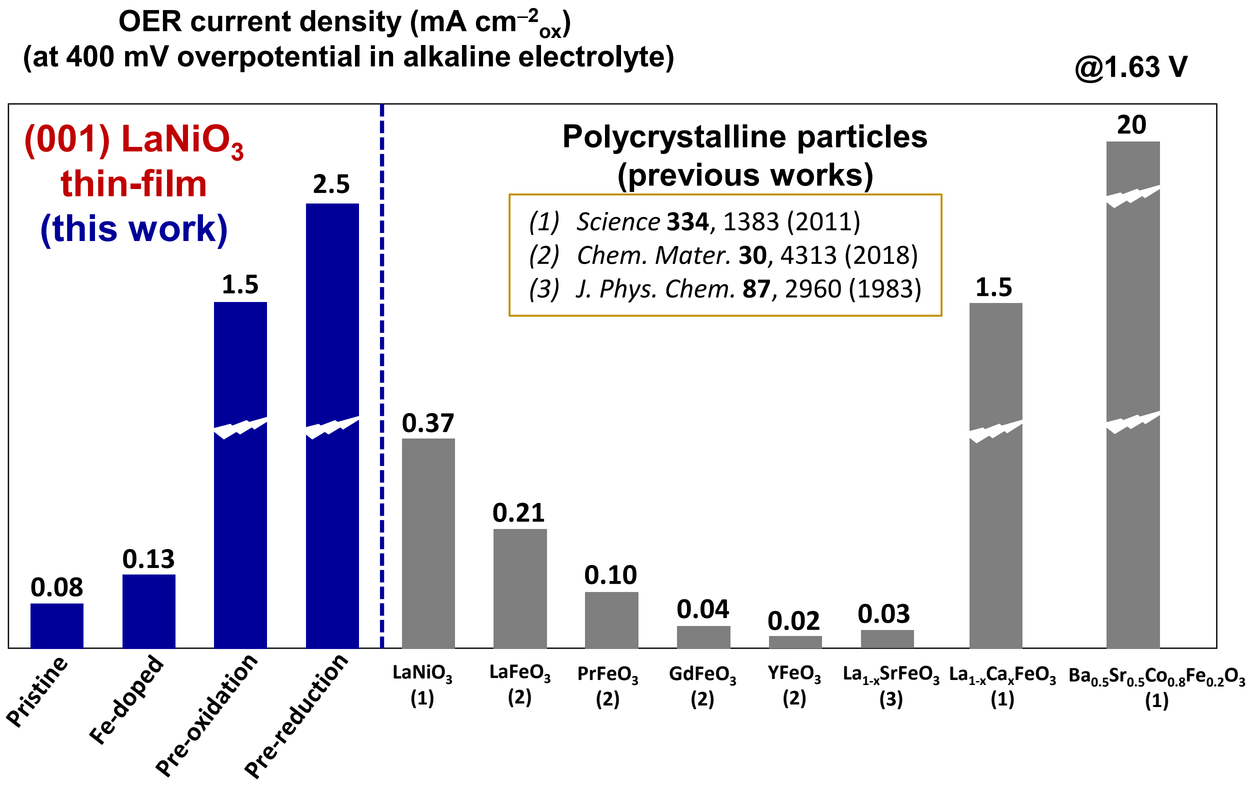

Supplementary Fig. 9. Comparison of OER activities. This bar graph provides a comparison between the OER activities of other Fe-containing perovskite oxides from previous works and our samples at 1.63 V *vs*. RHE. It is noted that all the OER activities reported in this work (blue bars) are of the (001) plane of $LaNiO_3$-based epitaxial thin films, while the results from previous works (gray bars) are based on polycrystalline particles with random crystallographic surface planes.

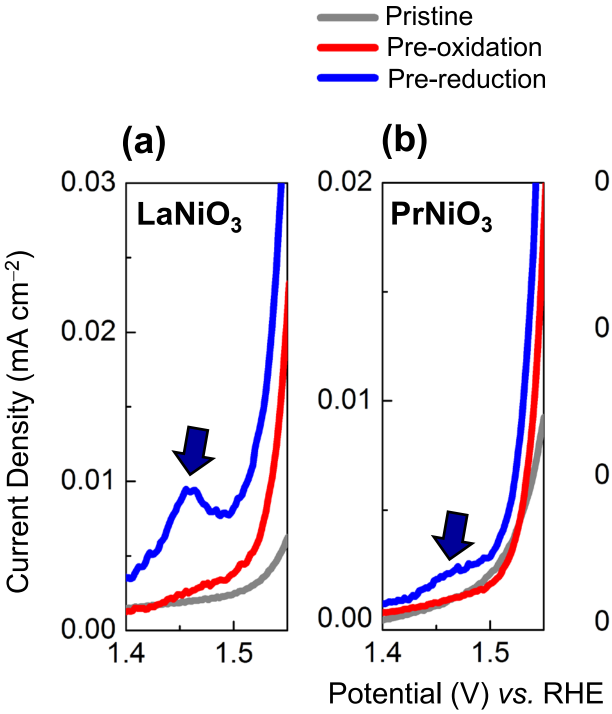

Supplementary Fig. 10. Appearance of a Ni^{2+}/Ni^{3+} anodic peak. In all three nickelates, (**a**) $LaNiO_3$, (**b**) $PrNiO_3$, and (**c**) $NdNiO_3$, Ni^{2+}/Ni^{3+} oxidation peak between 1.45 and 1.5 V *vs.* RHE is consistently identified in the thin-film samples subjected to the pre-reduction reaction, in contrast to the pristine and pre-oxidation samples. This supports the presence of NiO on the surface of the pre-reduction samples.

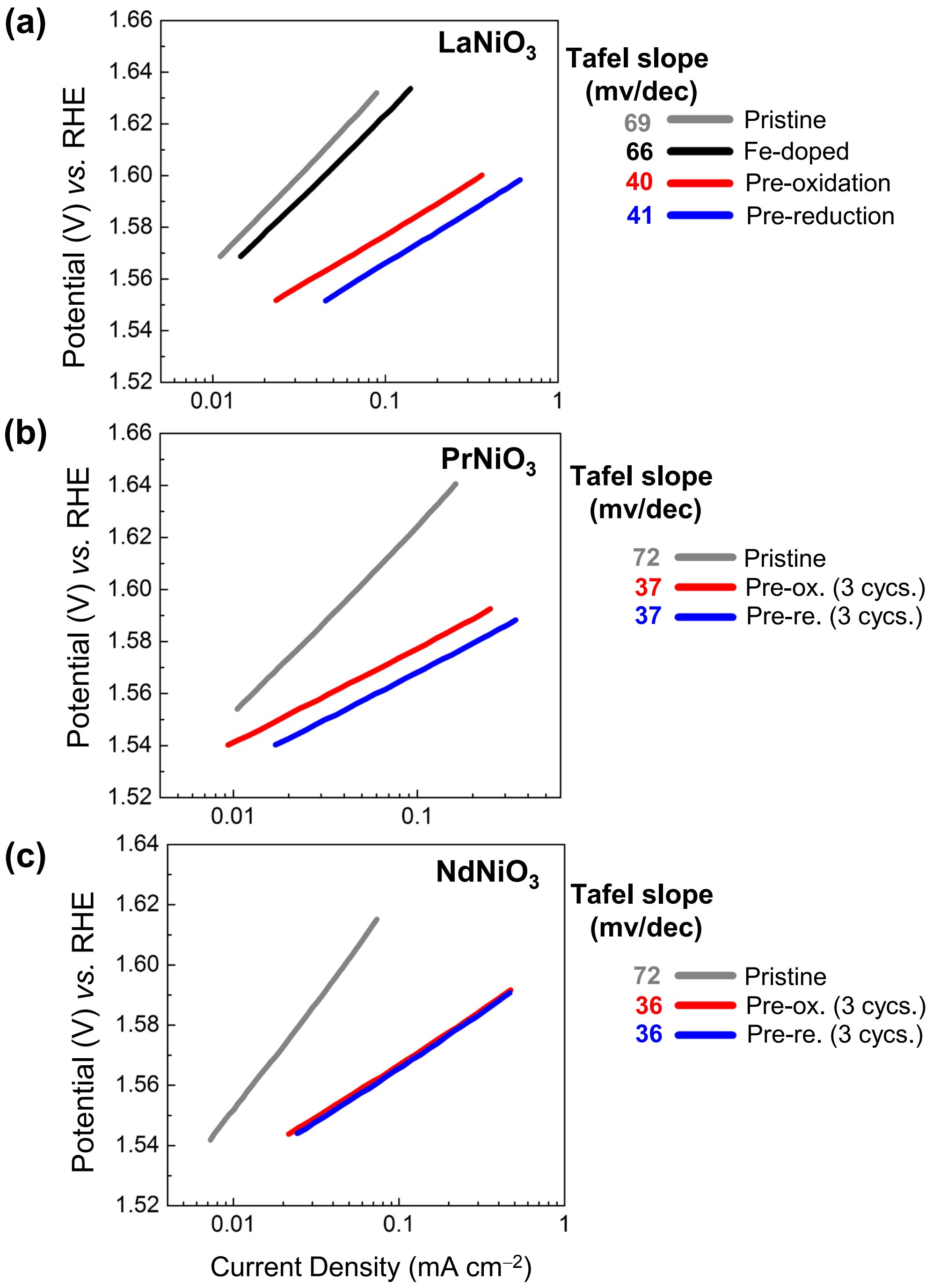

Supplementary Fig. 11. OER Tafel plots of thin-film samples. The linear correlations between the potential and the logarithmic current density are plotted for (**a**) $LaNiO_3$, (**b**) $PrNiO_3$, and (**c**) $NdNiO_3$ films. Comparatively lower values of Tafel slopes are acquired for the Fe-exchange samples in all three nickelate cases, showing approximately 30–40 mV/decade for the Fe-exchange samples, in contrast to ~70 mV/decade for the pristine samples. Therefore, from these Tafel plots, a substantial reduction in the activation barrier of the OER rate-limiting step in the pristine nickelates can be anticipated.

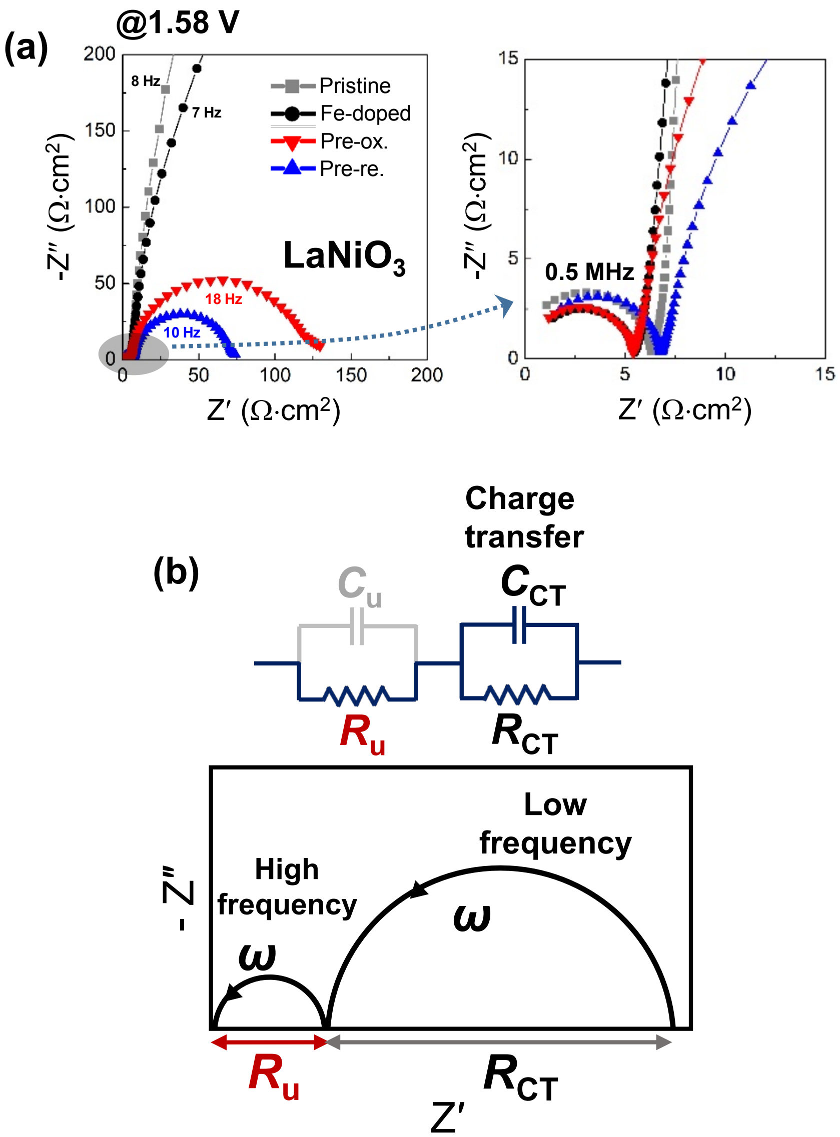

Supplementary Fig. 12. Complex-plane Nyquist plots acquired from the EIS and equivalent circuit for $LaNiO_3$ thin films. (**a**) The first semicircles in the Nyquist plots in our study stems from the uncompensated series resistance (R_u) between the working electrode and the Ag/AgCl reference electrode during the EIS, as explained in a recent article (*Chem. Mater.* **29**, 120–140 (2017)). The uncompensated series resistance, R_u, is given as $R_u = \frac{x}{\kappa A}$ (where x is the distance from the tip of the reference electrode to the working electrode, A is the area of the working electrode, and κ is the solution conductivity. As x in our measurement set-up is always nearly constant (approximately 3 mm), the R_u value, the diameter of the first arc, does not vary with the magnitude of the overpotential during the EIS. The cable and instrument capacitances and inductances are suggested to make a contribution to the appearance of the imaginary impedance, resulting in the semicircle formation at high frequencies (**b**) Schematic diagrams of the equivalent circuit and corresponding Nyquist plot for the nickelate thin films in this study are shown. Two *RC* elements in series are represented for the uncompensated component (R_uC_u) in the high-frequency range and the interface between the film and the electrolyte ($R_{CT}C_{CT}$) in the low-frequency range.

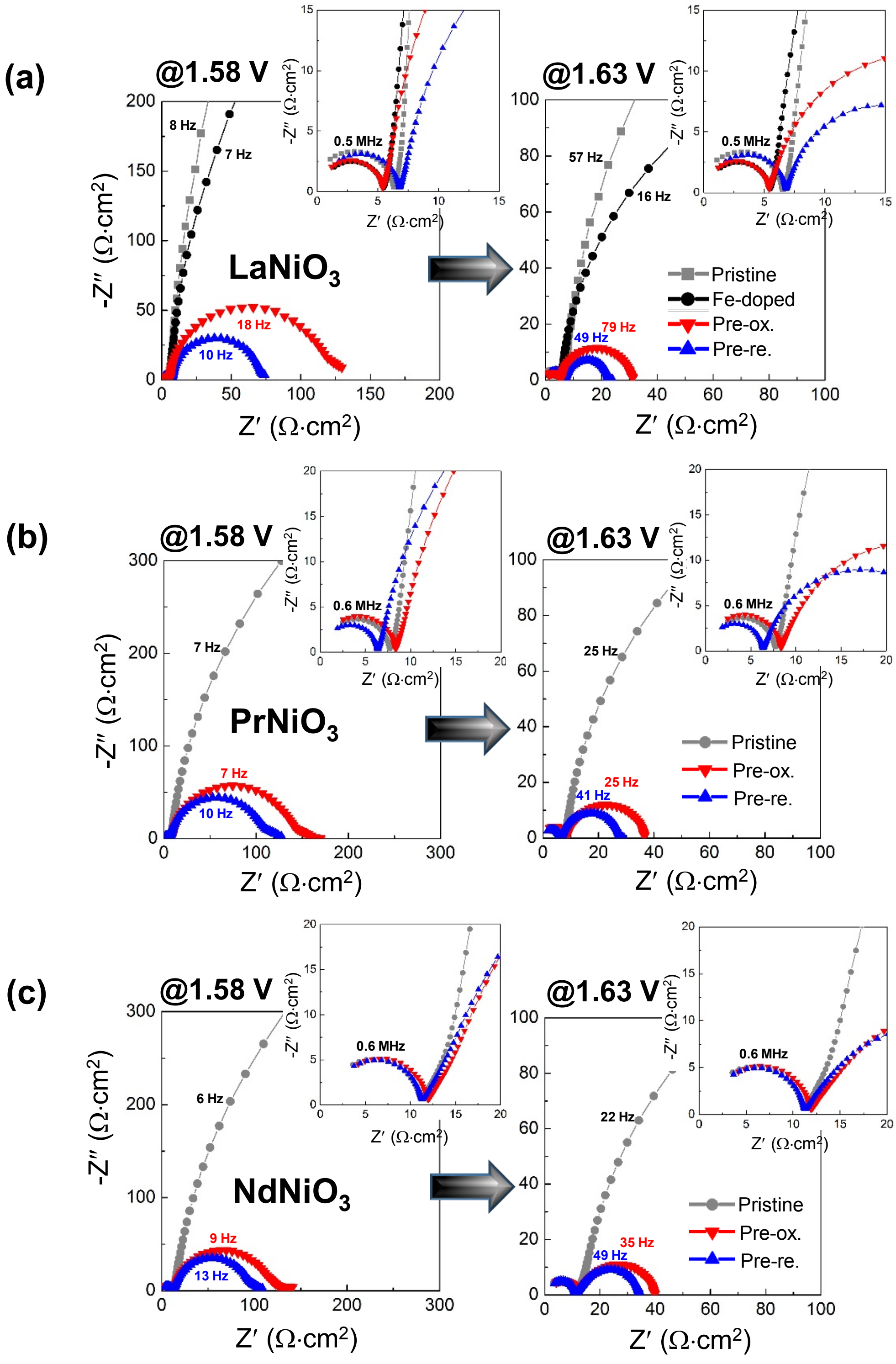

Supplementary Fig. 13. Complex-plane Nyquist plots acquired from the EIS. Impedance spectroscopy was carried our with application of under 1.58 V (left) and 1.63 V (right) *vs.* RHE in (**a**) $LaNiO_3$, (**b**) $PrNiO_3$ and (**c**) $NdNiO_3$ samples. Three important features are identified. First, the interface charge-transfer resistance, R_{CT} (the diameter of the low-frequency second semicircle) is remarkably low for the Fe-exchange samples following pre-reduction and pre-oxidation in all the three nickelates. Second, the R_{CT} values are substantially reduced at a higher overpotential, 1.63 V *vs*. RHE, showing a lower barrier for charge transfer. However, as the third feature, it is noted that the electrolyte resistance, R_E (the high-frequency first semicircle), is invariant, irrespective of the value of overpotential, as can be seen in the insets in each case.

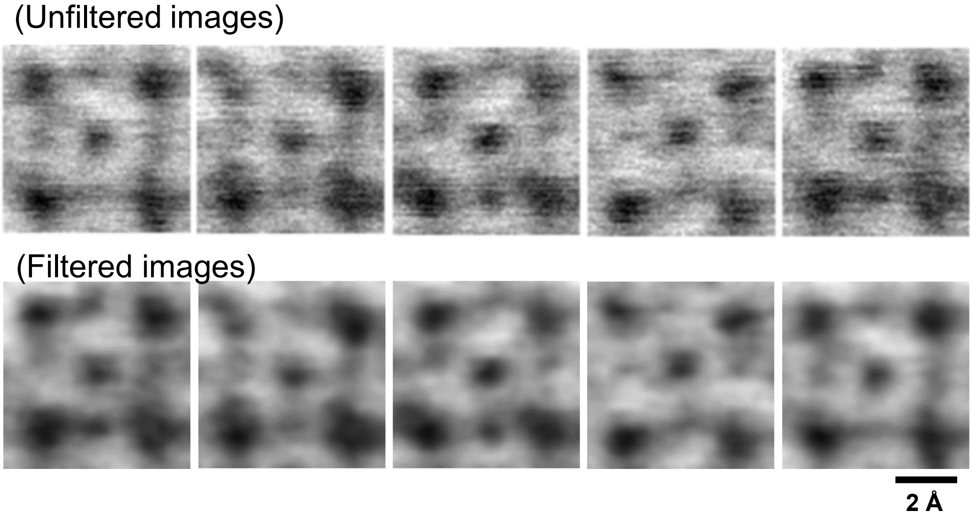

Supplementary Fig. 14. Comparison between unfiltered and filtered ABF images. Although the background noise is significantly reduced in the filtered images, the positions of atomic columns and the overall image features are not influenced by filtering.

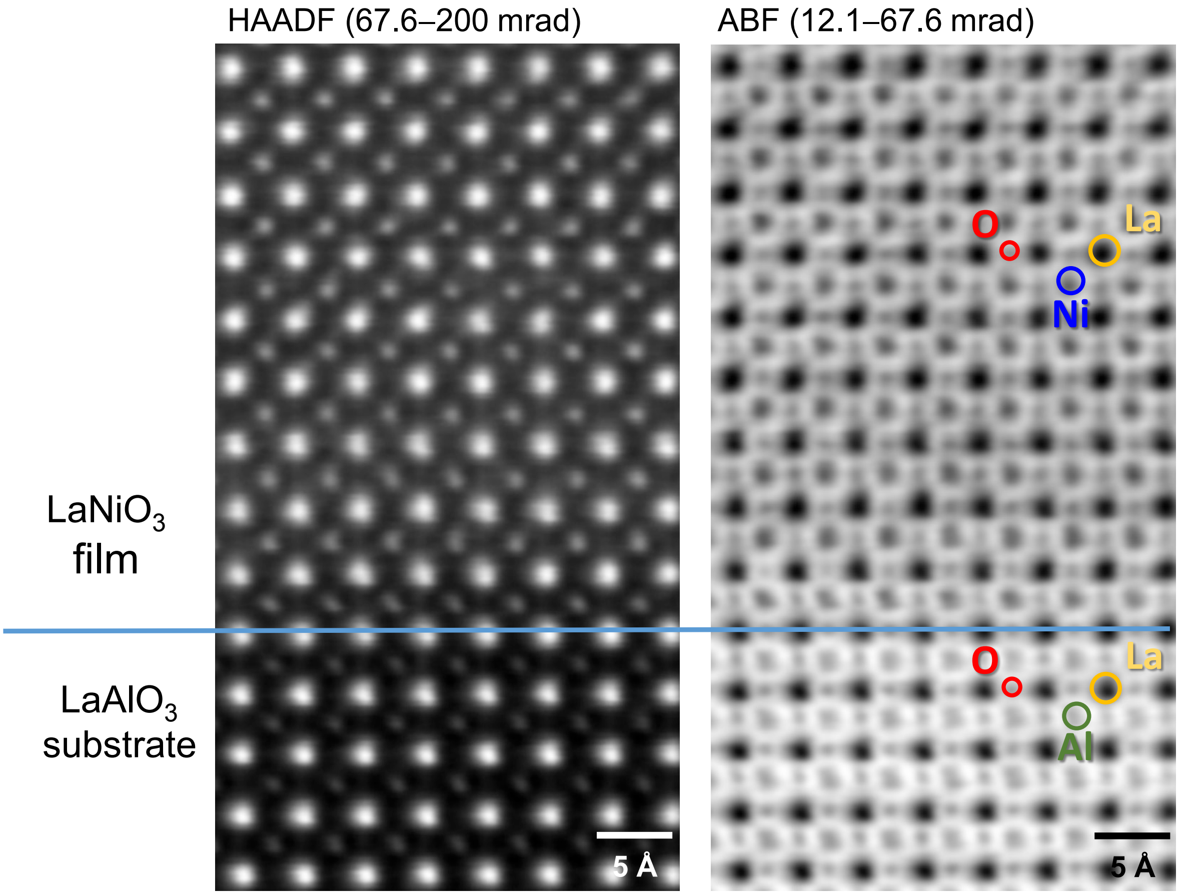

Supplementary Fig. 15. HAADF and ABF images simultaneously obtained from a $LaNiO_3$ epitaxial film on a $LaAlO_3$ substrate. As the typical collection semiangle for ABF imaging is 11−22 mrad, the dark-field signal is unavoidable when the collection angle is set to be 12.1−67.6 mrad in our study. However, sufficiently distinguishable contrast for the oxygen columns can be obtained under this angle condition.

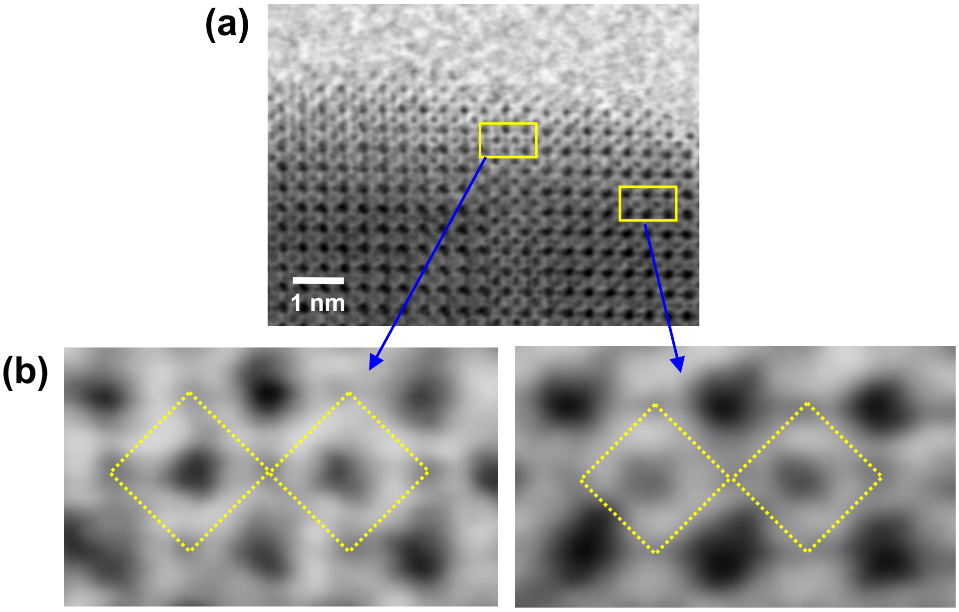

Supplementary Fig. 16. ABF images for the surface region of a pristine $LaNiO_3$ film.
(**a**) This ABF image was acquired from a film sample without electrochemical treatments.
(**b**) As shown in these two magnified images, undistorted oxygen octahedra (yellow diamonds) are verified at an atomic scale.

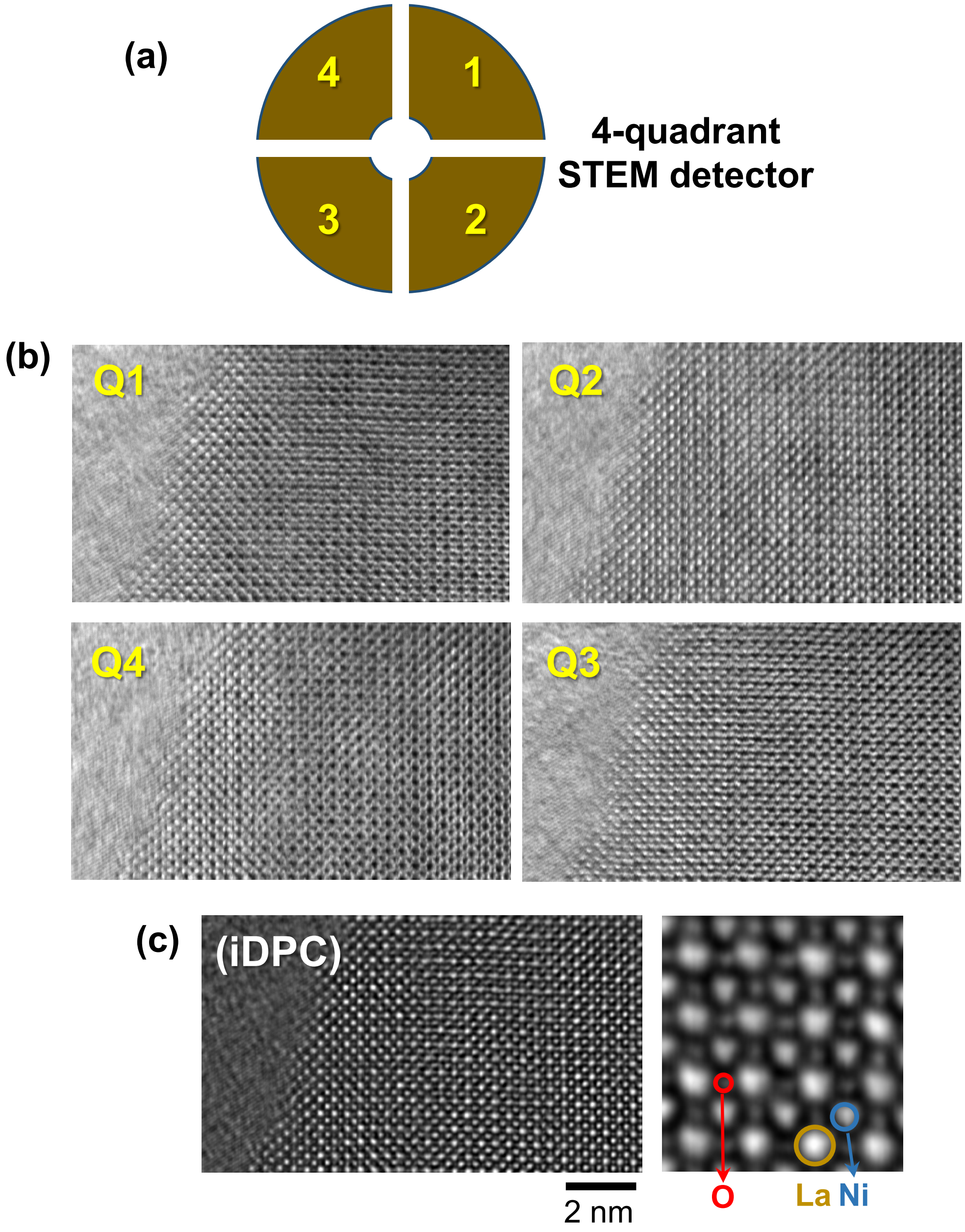

Supplementary Figure 17. Image construction using iDPC in $LaNiO_3$. (**a**) In this imaging mode, a four-quadrant segmented STEM detector is used, as illustrated in the figure. (**b**) A bright-field STEM image (Q1, Q2, Q3 and Q4) is captured in each quadrant of the detector. The differences between the two opposite quadrants (Q1–Q3 and Q2–Q4) are utilized to produce two differential phase-contrast images that are an excellent approximation of the center-of-mass component images. (**c**) An integrated differential phase-contrast image (iDPC) is finally acquired by integrating the two differential phase-contrast vector images. In addition to La and Ni, the O columns in $LaNiO_3$ are clearly visualized with high position accuracy, as demonstrated in the magnified image. More details can be found in references 30 and 31 of the main text.

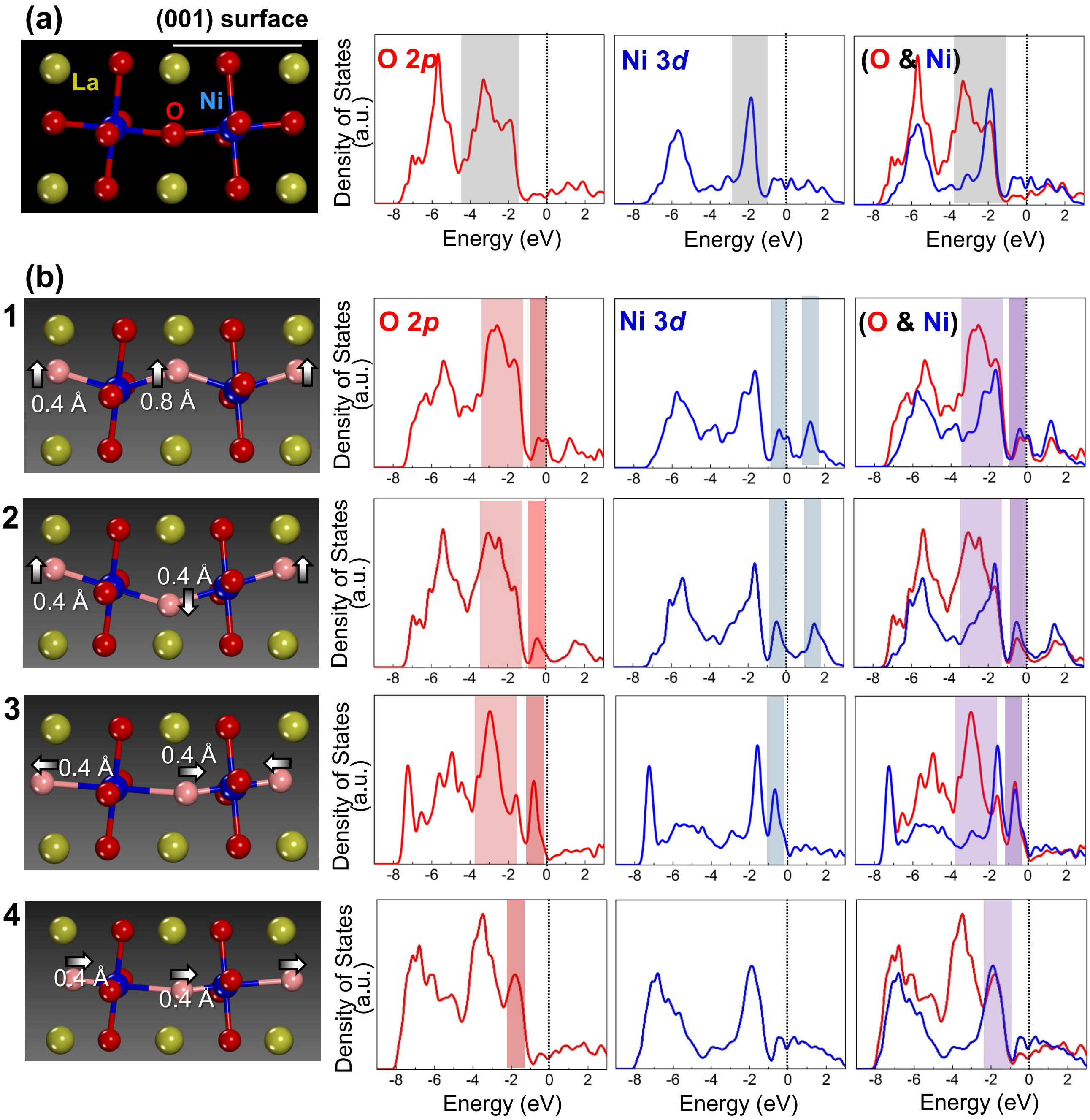

Supplementary Figure 18. DOS of (001) pristine $LaNiO_3$. A set of DOS plots for O 2*p*, Ni 3*d*, and metal/oxygen total states are provided for each case. (**a**) The optimized supercell of the (001) surface and its DOS were taken as references for comparison. A relatively high density of states in each DOS plot is denoted by a gray shadow. (**b**) Four independent cases for oxygen-octahedron distortion are exemplified, showing vertical (cases 1 and 2) and lateral (cases 3 and 4) displacements of oxygen. The significant DOS variations produced by the oxygen displacements are noted in each case. In particular, as indicated by dark shadows, there is a substantial increase in the DOS near the Fermi level despite the absence of Fe. This appears to have a beneficial effect permitting easier charge transfer between oxygen and metals.

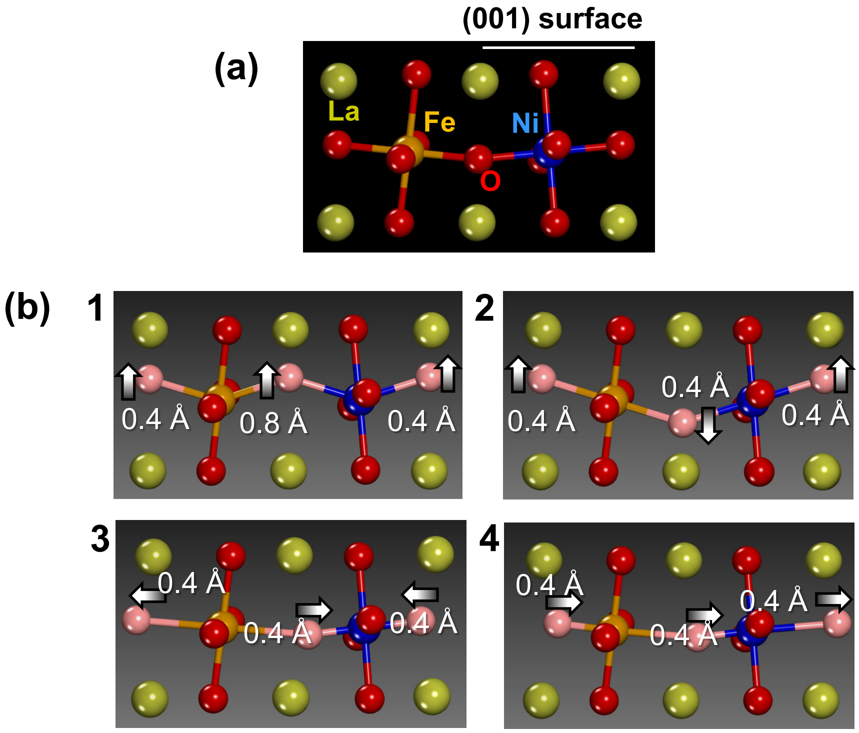

Supplementary Figure 19. Indication of oxygen displacements in the supercells. (**a**) The (001) surface region of a supercell for the DFT calculations is illustrated. (**b**) As denoted by white arrows, 0.4-Å displacement from the initial position is applied to the oxygen atoms except one in supercell #1 (0.8 Å).

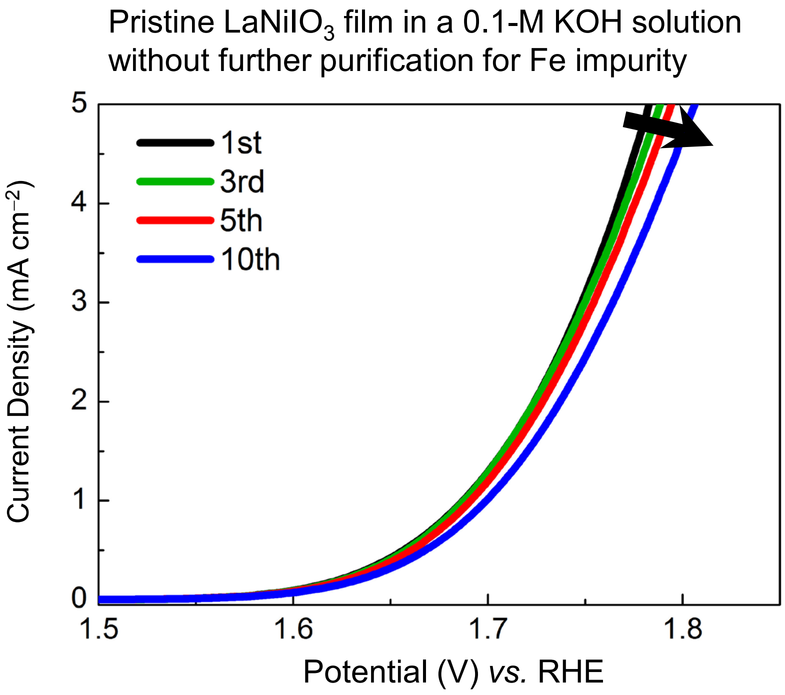

Supplementary Fig. 20. Plot for the OER current densities of a pristine $LaNiO_3$ film. Our KOH electrolyte solutions were prepared by using the Milli-Q water (18.2 MΩ·cm) and KOH pellets (Sigma Aldrich, 99.99%) to achieve sufficiently high purity. The OER current density continuously decreases, as shown in this plot, directly supporting the absence of an Fe-impurity effect in the electrolyte. Cyclic potential was applied for the OER current measurements in a range from 1.23 to 1.83 V *vs.* RHE.

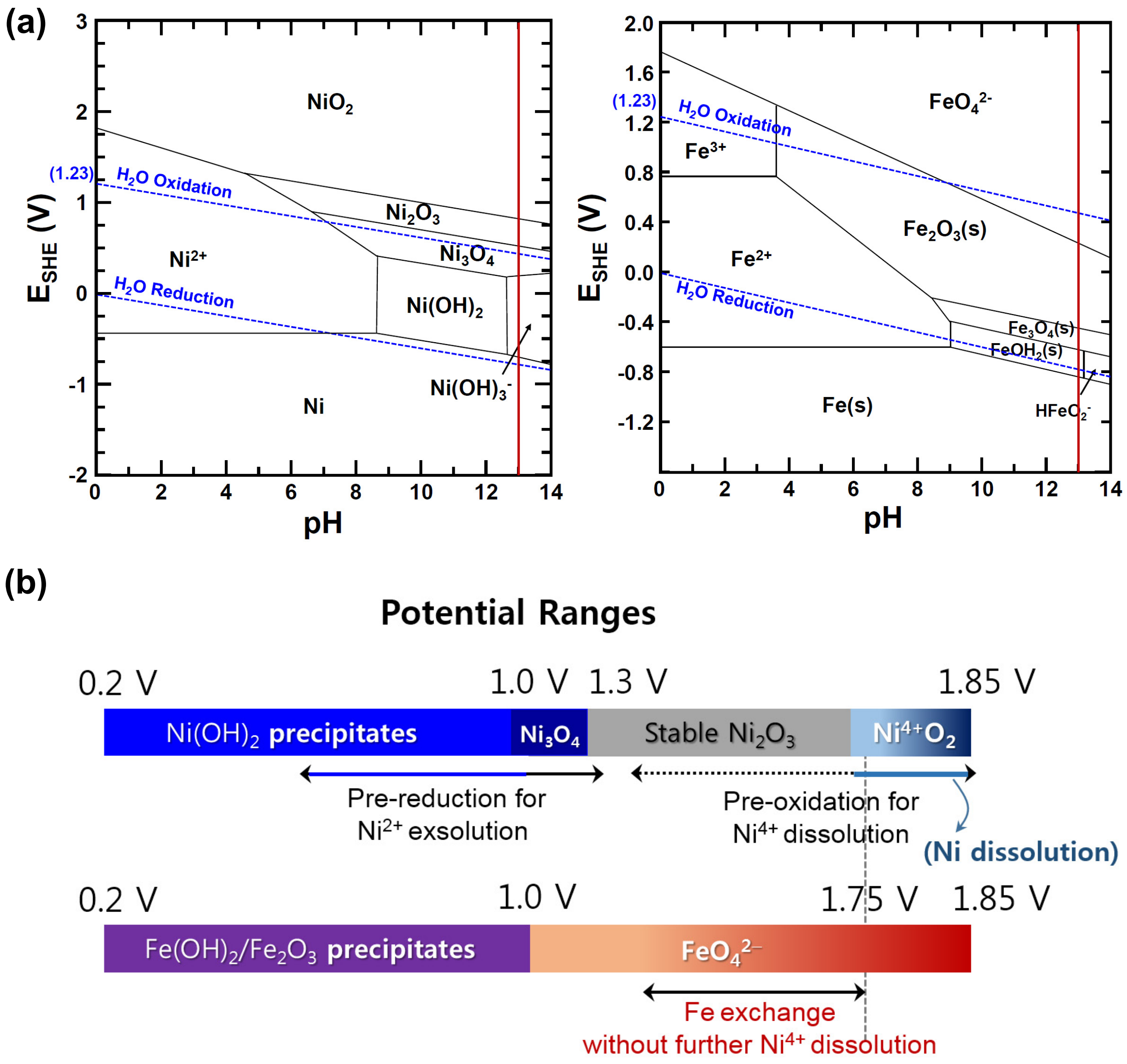

Supplementary Fig. 21. Pourbaix diagrams and relevant potential ranges for reactions. (**a**) The Pourabix diagrams of Ni (left) and Fe (right) are provided to show the stable forms of Ni and Fe at pH =13 (red vertical lines) as a function of the potential. Theses diagrams were redrawn from recent reports (Huang, L.-F. *et al*. *J. Phys. Chem. C* **121**, 9782–9789 (2017) with permission (copyright 2017 American Chemical Society) and Tolouei, R. *et al*. *Phys. Chem. Chem. Phys.* **18**, 19637–19646 (2016) with permission (copyright 2016 Royal Society of Chemistry)) (**b**) This illustration is based on the two Pourbaix diagrams of Ni and Fe. Most of the Ni-vacancy formation takes place between 1.70 V and 1.85 V where substantial Ni dissolution (as Ni^{4+}) from the $LaNiO_3$ film occurs, although a potential range from 1.27 to 1.85 V is selected for the pre-oxidation reaction. In addition, the potential range for the Fe exchange was from 1.27 to 1.75 V to avoid further Ni vacancy formation via Ni dissolution. As clarified in the Pourabix diagrams of Fe in (a), under a weak potential (<1.0 V), most Fe is present as solid-state $Fe(OH)_2$ or Fe_2O_3 precipitates in the solution, thereby causing Fe exchange to fail. Consequently, the application of sufficient anodic potential (>1.0 V *vs*. RHE) is necessary for the Fe exchange.

Supplementary Note 1

Formation of Ni vacancies during the pre-reduction reaction

When a potential far below 1.2 V is applied to $LaNiO_3$ in an alkaline solution (pH = 13), Ni^{3+} cations in the $LaNiO_3$ lattice begin to be reduced as Ni^{2+}, as shown in the Ni Pourbaix diagram (see the following figure redrawn from a recent study (Huang, L.-F. *et al. J. Phys. Chem. C* **121**, 9782–9789 (2017) with permission. Copyright 2017 American chemical Society).

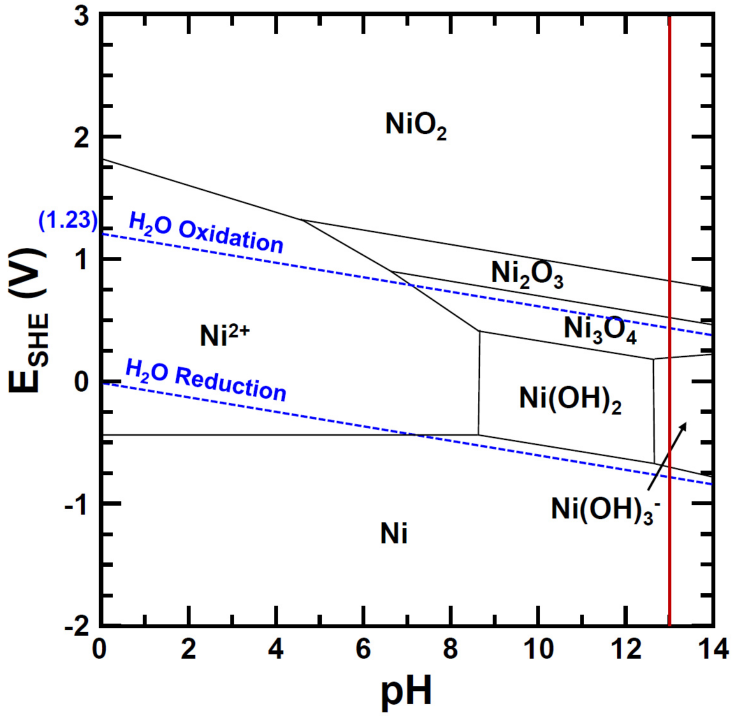

< Ni Pourbaix diagram >

Because the ionic radius of Ni^{2+} in the octahedral interstitial (r = 69 pm) is more than 20% larger than that of Ni^{3+} (r = 56 pm), a considerably large compressive strain energy drives Ni^{2+} cations to exsolve out of the lattice, resulting in the formation of Ni vacancies in the lattice and simultaneously $Ni(OH)_2$ precipitates on the surface of the $LaNiO_3$ film (Supplementary Fig. 3). In contrast to the environment during the pre-oxidation reaction, oxygen vacancies do not readily form during the pre-reduction. Therefore, the effectively negative charge of Ni vacancies should be compensated by other positively charged defects rather than by oxygen vacancies

As readily recognized in the following figure showing the density of states (DOS) of $LaNiO_3$ obtained by the DFT calculation, strong hybridization between the Ni 3*d* orbitals and O 2*p* orbitals is demonstrated in the valence band below the Fermi level (E = 0 eV in the DOS).

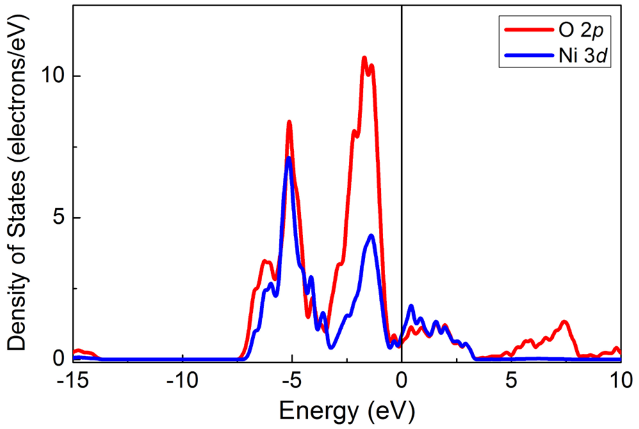

< Density of states of $LaNiO_3$ >

Subsequently, based on this *high degree of covalency* in Ni–O bonding, an energetically favorable method of Ni^{3+} reduction to Ni^{2+} is electron transfer from oxygen to Ni, which results in the formation of electron holes in the oxygen 2*p* orbitals in the valence band. DFT calculation for an electronic population analysis was carried out in order to examine the charge variation of neighboring oxygens. As indicated in the following figure, a noticeable change of the Mulliken charge of oxygens adjacent to a Ni vacancy is identified. The variation from –0.65e to –0.63e directly denotes the loss of electrons and consequently the formation of holes. It is worthwhile to note that, due to the high degree of Ni–O covalency, this Mulliken charge variation is confined to the nearest oxygens to the Ni vacancy. Therefore, the electron holes created by the electrochemical reduction appear to be fairly localized around Ni vacancies.

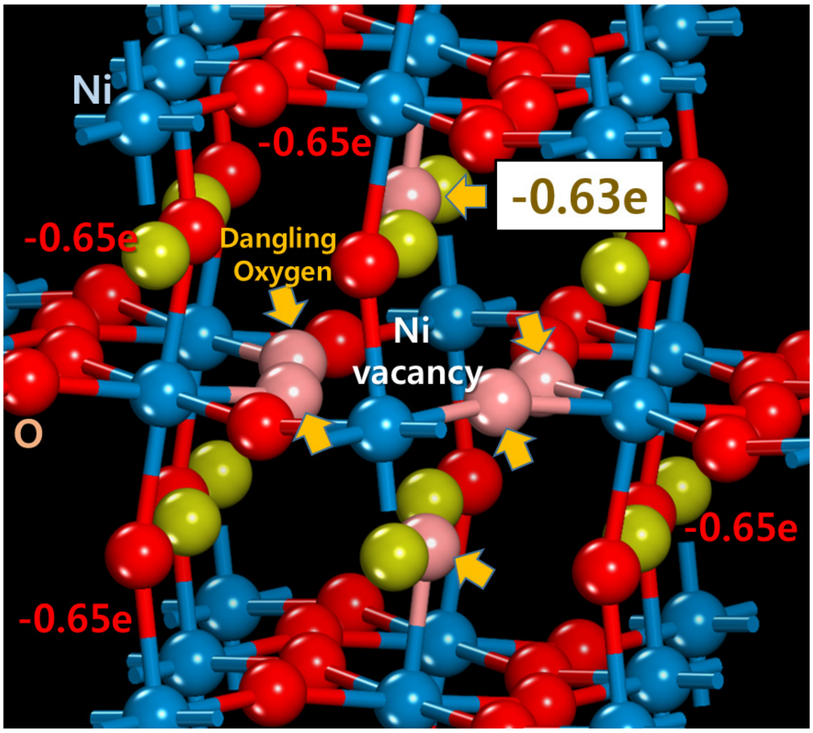

< Mulliken charge values of oxygens around a Ni vacancy in $LaNiO_3$ >

On the basis of this DFT electronic population analysis, the overall charge neutrality during the formation of Ni vacancies via the electrochemical pre-reduction reaction can be reasonably suggested to be $V_{Ni}''' + 3O_O^{\bullet}$ (where $O_O^{\bullet}$ denotes electron holes ($h^{\bullet}$) confined to the oxygens adjacent to the Ni vacancy). In addition, as a number of K^+ ions are present in the KOH electrolyte solution, their contribution to the charge neutrality at the film surface should not be excluded. Therefore, $V_{Ni}''' + aK^{\bullet}_{surf}$ (adsorbed on the surface) + $bO_O^{\bullet}$ (where $a + b = 3$) is another possible defect chemical consideration to satisfy the charge neutral condition. Note that the effective charge of the Ni vacancy is assumed to be −3, as the trivalent state of Ni is a stable condition in the $LaNiO_3$ lattice. Through the Fe exchange under the anodic condition, some Ni vacancies can be filled with Fe^{3+} cations and electrons from OH^- in the electrolyte solution also can be transferred to annihilate the holes for charge neutrality (for example, $(1-x)V_{Ni}''' + xFe_{Ni} + (3-x)O_O^{\bullet} + xO_O$).

Supplementary Note 2

Formation of Ni vacancies during the pre-oxidation reaction

As can be seen in the Ni Pourbaix diagram, the valence state of Ni^{3+} becomes higher to be Ni^{4+} as NiO_2, when more than 1.7 V *vs*. RHE for the anodic potential is applied at pH =13. Based on our experiments (as already shown in Supplementary Fig. 4), it appears that Ni^{4+} can be dissolved in a strong alkaline solution during the pre-OER treatment at high overpotentials over 1.7 V. In fact, we could not observe any noticeable Ni dissolution and subsequent lattice amorphization when the overpotential range was confined between 1.2 V and 1.7 during the pre-OER. As a result, it is believed that the application of sufficiently high overpotential is essential to efficiently create Ni vacancies.

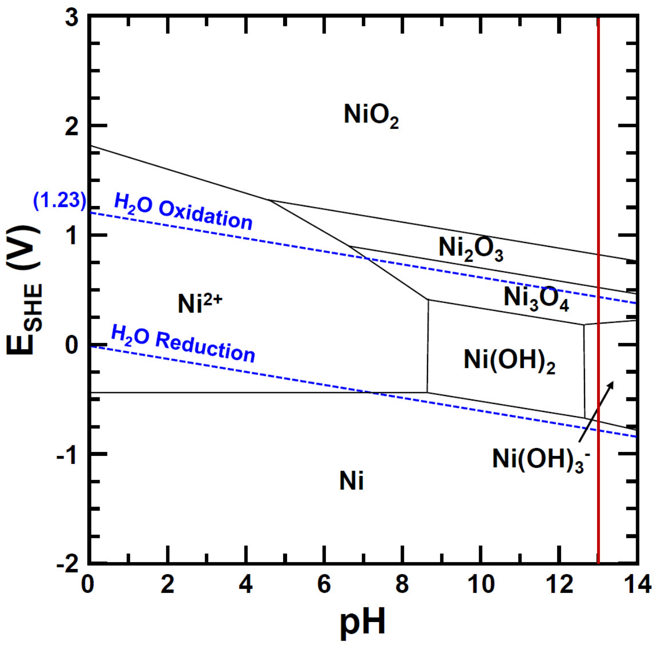

< Ni Pourbaix diagram >

Because the Ni vacancies are negatively charged defects, the formation of other point defects with an effectively positive charge should be accompanied to satisfy the overall charge neutrality condition. Under a strong anodic environment, oxygen anions in the lattice can be involved in the OER, resulting in the formation of oxygen vacancies. Consequently, the Schottky-type cation–anion vacancy pairs ($2V_{Ni}''' + 3V_O^{\bullet\bullet}$) are expected to form during the pre-OER. If Fe cations are sufficiently provided, some Ni vacancies can be filled with the cations and subsequently some oxygen vacancies are also filled with OH^- anions from the KOH solution for the charge neutrality (for example, $(2-x)V_{Ni}''' + xFe_{Ni} + (3-x)V_O^{\bullet\bullet} + xOH_O^{\bullet}$ in the Kröger–Vink notation).
